# Genome-wide identification and differential analysis of translational initiation

**DOI:** 10.1038/s41467-017-01981-8

**Published:** 2017-11-23

**Authors:** Peng Zhang, Dandan He, Yi Xu, Jiakai Hou, Bih-Fang Pan, Yunfei Wang, Tao Liu, Christel M. Davis, Erik A. Ehli, Lin Tan, Feng Zhou, Jian Hu, Yonghao Yu, Xi Chen, Tuan M. Nguyen, Jeffrey M. Rosen, David H. Hawke, Zhe Ji, Yiwen Chen

**Affiliations:** 1 Department of Bioinformatics and Computational Biology, The University of Texas MD Anderson Cancer Center, Houston, TX 77030 USA; 2 Proteomics and Metabolomics Facility, and Department of Systems Biology, The University of Texas MD Anderson Cancer Center, Houston, TX 77030 USA; 3 Department of Biochemistry, State University of New York at Buffalo, Buffalo, NY 14203 USA; 4 Avera Institute for Human Genetics, Sioux Falls, SD 57108 USA; 5 Liver Cancer Institute, Zhongshan Hospital, Key Laboratory of Carcinogenesis and Cancer Invasion, Minister of Education, and Institutes of Biomedical Sciences, Fudan University, Shanghai, 200032 China; 6 Department of Cancer Biology, The University of Texas MD Anderson Cancer Center, Houston, TX 77054 USA; 7 Department of Biochemistry, The University of Texas Southwestern Medical Center, Dallas, TX 75390 USA; 8 Department of Molecular and Cellular Biology, Baylor College of Medicine, Houston, TX 77030 USA; 9 Program in Translational Biology and Molecular Medicine, Baylor College of Medicine, Houston, TX 77030 USA; 10 Department of Biological Chemistry and Molecular and Pharmacology, Harvard Medical School, Boston, MA 02115 USA; 11 Broad Institute of MIT and Harvard, Cambridge, MA 02142 USA

## Abstract

Translation is principally regulated at the initiation stage. The development of the translation initiation (TI) sequencing (TI-seq) technique has enabled the global mapping of TIs and revealed unanticipated complex translational landscapes in metazoans. Despite the wide adoption of TI-seq, there is no computational tool currently available for analyzing TI-seq data. To fill this gap, we develop a comprehensive toolkit named Ribo-TISH, which allows for detecting and quantitatively comparing TIs across conditions from TI-seq data. Ribo-TISH can also predict novel open reading frames (ORFs) from regular ribosome profiling (rRibo-seq) data and outperform several established methods in both computational efficiency and prediction accuracy. Applied to published TI-seq/rRibo-seq data sets, Ribo-TISH uncovers a novel signature of elevated mitochondrial translation during amino-acid deprivation and predicts novel ORFs in 5′UTRs, long noncoding RNAs, and introns. These successful applications demonstrate the power of Ribo-TISH in extracting biological insights from TI-seq/rRibo-seq data.

## Abstract (web summary)

Translation initiation sequencing (TI-seq) has revealed unexpected diversity in protein isoforms. Here, Zhang et al. present Ribo-TISH, a computational toolkit that can detect and compare TIs across conditions and improve open reading frame prediction from different types of ribosome profiling data.

## Introduction

Translation is an essential step of gene expression. It is tightly controlled^1^ and is crucial to numerous developmental^2^ and physiological processes^3, 4^, such as early embryogenesis^2^ and stress responses^4, 5^, where translational control of the pre-existing mRNAs can change the final protein abundance more rapidly than the synthesis of new mRNAs. The dysregulation of translation is associated with many diseases, such as anemia^6^, neurological disorders^7^, and cancer^8^.

The development of the ribosome profiling (ribo-seq) technique has enabled the high-resolution measurement of translation on a genome-wide scale^1–3^. The basic procedure of ribo-seq is to perform deep sequencing of the DNA libraries converted from the ribosome-protected mRNA fragments (RPFs, also termed ribosome footprints) that are generated by RNase digestion, to determine the occupancy of translating ribosomes on a given mRNA. There are several variations of the ribo-seq technique that use different translation inhibitors^4–6^. Regular ribo-seq (rRibo-seq) utilizes cycloheximide (CHX)^4^, a translation elongation inhibitor, to freeze all translating ribosomes. Recent studies using CHX-based rRibo-seq revealed pervasive translation in the genomic regions that are beyond the annotated protein-coding regions^9–13^. These newly discovered translated regions not only include small open reading frames (smORFs, ≤100 amino acids) in intragenic regions of protein-coding genes (PCGs), such as those in the 5′ untranslated region (5′UTR; upstream ORFs, uORFs) or 3′UTR (downstream ORFs, dORFs) but also include the smORFs within long noncoding RNAs (lncRNAs)^14, 15^, which were not expected to encode any sizable proteins. The human genome encodes over 15,000 lncRNA genes. Based on rRibo-seq data, it has been estimated that ~40% of lncRNA genes may contain translated smORFs^12^. A few of the smORFs within lncRNAs have been shown to play essential developmental or physiological roles in evolutionarily distant species^16–19^.

Translation is largely regulated at the initiation stage^20^. Therefore, elucidating the mechanism and regulation of translation initiation (TI) is fundamental to our understanding of translational regulation. The use of the translation inhibitor lactimidomycin (LTM)^21^ or harringtonine (Harr)^22^, which has a much stronger effect for capturing initiating ribosomes, allows for the global mapping of TI sites (TISs) by sequencing (TI-seq). When LTM is used sequentially with puromycin, the corresponding TI-seq experiment, known as quantitative TI-seq (QTI-seq), enables a quantitative comparison of TI under different conditions^23^. In eukaryotes, the first AUG start codon that the ribosome encounters is most often selected to initiate translation. However, many alternative TISs downstream and upstream of the first AUG have been revealed^24, 25^. The use of alternative TISs is an important mechanism for creating protein isoform diversity^26–30^ at the translational level, whereby an N-terminal truncated or extended protein variant can be generated. It was estimated that 20% of the protein N termini identified in mouse and human cells by mass spectrometry may correspond to alternative TI (aTI)^31^, many of which are initiated at near-cognate non-AUG start codons^32^. In comparison with the CHX-based rRibo-seq, the TI-seq/QTI-seq has proven to be a more powerful technique in aiding the discovery and quantitation of aTI events^21, 23, 31, 33^, and is thus a critical tool for discovering novel translational protein isoforms resulting from aTI (aTI isoforms) and for elucidating the function and mechanism of TI.

Despite the broad applicability of the TI-seq/QTI-seq technique, it remains challenging to distinguish the true signal from noise and to extract useful information from TI-seq/QTI-seq data. Computational methods have been developed for the analysis of rRibo-seq data^12, 34–44^. However, there is no statistically principled and computationally efficient tool available for detecting and quantitatively comparing TIs under different conditions from TI-seq/QTI-seq data. To fill this gap, we develop a computational toolkit named ribo-seq data-driven TIS hunter (Ribo-TISH). Aside from the analysis of TI-seq/QTI-seq data, it can predict ORFs from rRibo-seq data and outperform several established methods. When applied to published data sets, Ribo-TISH reveals an unexpected role of elevated mitochondrial translation in cellular stress response induced by amino-acid deprivation and uncovers novel ORFs beyond the annotated protein-coding regions, demonstrating its utility in extracting new insights from TI-seq/rRibo-seq data.

## Results

### An overview of Ribo-TISH

**Fig. 1:**
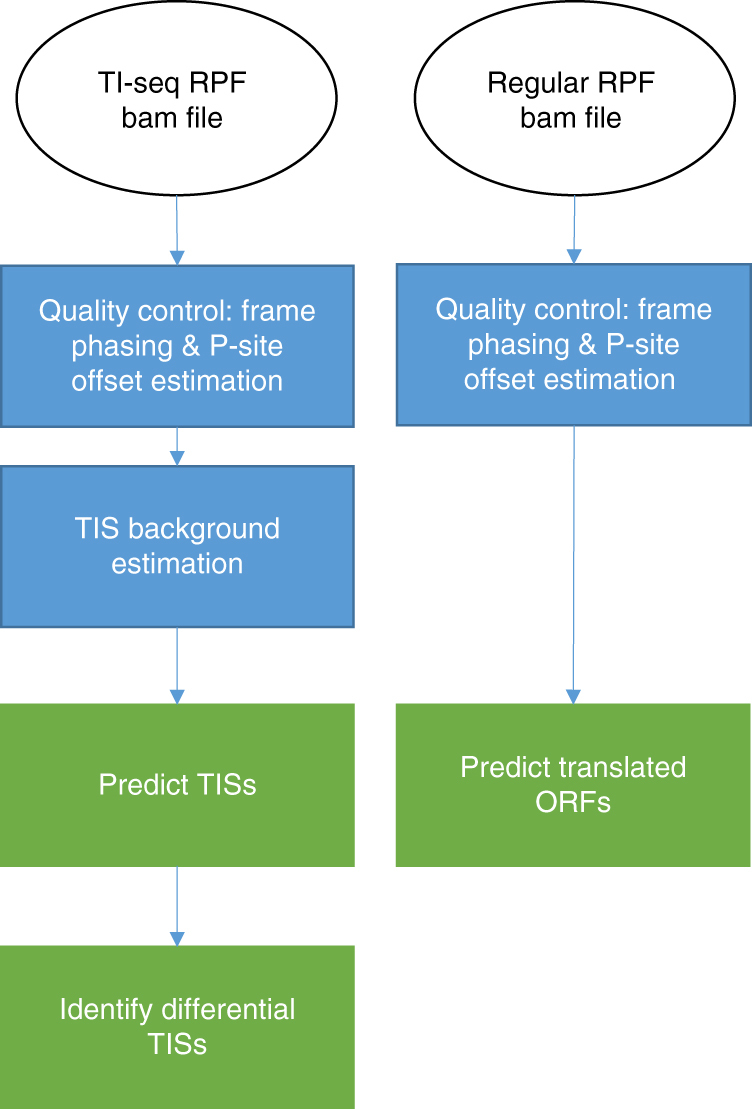
A schematic overview of Ribo-TISH. Ribo-TISH starts from quality control of the aligned sequencing data to identifying and differential analysis of translation initiations from TI-seq/QTI-seq data, and to predicting actively translated ORFs from rRibo-seq data

Ribo-TISH was designed as a comprehensive toolkit for identifying and quantitatively comparing genome-wide TIs from TI-seq/QTI-seq data and for predicting putative ORFs from CHX-based rRibo-seq data. It uses as input the BAM alignment files generated from TI-seq or rRibo-seq raw data (Fig. 1). Based on the alignment files, Ribo-TISH provides a set of metrics/profiles to evaluate data quality (Fig. 1). These quality control (QC) metrics can identify the potential problems in the data for experimental optimization and can be used to filter out data of low quality for downstream analysis. Furthermore, Ribo-TISH utilizes data-driven methods to identify potential TISs from TI-seq/QTI-seq data, determine which TISs show differential initiation rates under different conditions from QTI-seq data, and predict actively translated ORFs from rRibo-seq data (Fig. 1).

### Quality control of TI-seq and rRibo-seq data

**Fig. 2:**
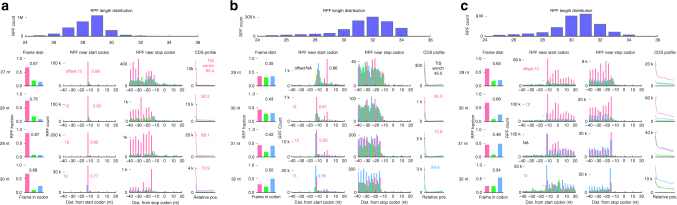
Quality control of TI-seq and rRibo-seq data. Quality control with Ribo-TISH for two TI-seq data sets generated using **a** LTM or **b** Harr, and **c** one rRibo-seq data set generated using CHX. Upper panel: length distribution of RPFs uniquely mapped to annotated protein-coding regions. Lower panel: different quality profiles/metrics for RPFs uniquely mapped to annotated protein-coding regions. The data corresponding to the first, second and third reading frame are colored in pink, light green and sky blue, respectively. Each row shows the RPFs with indicated length. Column 1: distribution of RPF 5′ end across three reading frames in all annotated codons; showing the fraction of RPF counts from dominant reading frame (*f*_d_). Column 2: distribution of RPF 5′ end count near annotated TISs; showing estimated P-site offset and the ratio (*f*_t_) between the RPF counts at the annotated TISs and the sum of the RPF counts near the annotated TISs (from −1 to +1 relative to the annotated TISs) after P-site offset correction. Column 3: distribution of RPF 5′ end count near annotated stop codon. Column 4: RPF count profile throughout protein-coding regions across three reading frames; showing TIS enrichment score for TI-seq data

In a TI-seq or rRibo-seq experiment, the size of RPFs recovered from gel electrophoresis for sequencing are typically around 30 nucleotides (nts). Ribo-TISH summarizes the distribution of RPF lengths using the sequenced RPFs that are mapped to the annotated PCGs and provides a measure of the size selection quality. As illustrated in the case of two TI-seq experiments using LTM^21^ (Fig. 2a) or Harr^22^ (Fig. 2b), and one rRibo-seq experiment using CHX^45^ (Fig. 2c), the RPF length distribution can vary across different experimental conditions. Based on the RPF length distribution, Ribo-TISH further provides several QC metrics/profiles to evaluate the quality of RPFs with different lengths.

The first category of QC profile/metric is the distribution of RPF counts across three reading frames and the fraction of the RPF counts in the dominant frame (*f*_d_) within the annotated PCGs at different RPF lengths (Fig. 2). Data of better quality are expected to have a higher fraction of RPF counts in the actively translated reading frame than the other two frames, i.e., a larger *f*_d_. The data quality can vary for RPFs with different lengths (e.g., Fig. 2c, smaller *f*_d_ for the RPFs with 31 nts). For rRibo-seq data, to ensure the inclusion of RPFs with excellent sub-codon frame phasing or 3-nt periodicity for analysis, Ribo-TISH keeps only the RPF lengths at which *f*_d_ is above 0.5 (i.e., the total number of reads in the dominant reading frame is higher than the total number of reads in the other two reading frames) for downstream analysis under the default setting (Methods). Because the magnitude of 3-nt periodicity can vary between different ribo-seq data sets and the default threshold of 0.5 may be too stringent for some data sets, Ribo-TISH allows users to define a customized threshold of *f*_d_ for different data sets.

The second category of QC profile/metric is the meta-gene profile of the RPF count near the annotated TISs and translation termination sites. A TI-seq or rRibo-seq data set with good quality is expected to show a sharp increase in RPF count near annotated TIS sites and a clear reduction near annotated translation termination sites. The ribosomal P-site is where the tRNA carrying the growing peptide chain is formed on the ribosome. The P-site is also the entry point for the first aminoacyl tRNA, where the canonical initiating Met-tRNA_i_^Met^ is base-paired with the AUG start codon. The P-site is usually internal to the sequenced RPFs, and Ribo-TISH determines the distance between the P-site and the 5′ end of the sequenced RPFs (i.e., the P-site offset) according to the meta-gene profile of the 5′ end of the RPFs with respect to the annotated TISs (Methods). The P-site offset can vary for RPFs with different lengths. Taking a Harr-based TI-seq data set (Fig. 2b) as an example, the P-site offset is 12 nts for the RPFs with length of 30 and 31 nts; whereas, the offset is 13 nts for the RPFs with the length of 32 nts. After P-site offset correction, Ribo-TISH calculates the ratio (*f*_t_) between the RPF counts at the annotated TISs and the sum of the RPF counts near the annotated TISs (from −1 to +1 relative to the annotated TISs) at different RPF lengths for TI-seq data. Similar to the case of rRibo-seq data, Ribo-TISH keeps the RPF lengths at which *f*_t_ is above a user-definable threshold (default 0.5) for downstream analysis.

To better quantify the enrichment of the RPF count at the TISs vs. the whole CDS region, the third category of QC metric/profile used by Ribo-TISH is the meta-gene profile of the RPF count across the whole CDS of the annotated PCGs and the TIS enrichment score after P-site offset correction, which is the ratio between the RPF count at the annotated TISs and the mean RPF count across the whole CDS region in the same reading frame. This metric is designed for QC of TI-seq data. The higher the TIS enrichment score, the more the initiating ribosomes is stalled and the better the quality of the TI-seq data. The meta-gene profile and TIS enrichment score are also provided for RPFs with different lengths.

The RPF density profile across the CDS region is not only shaped by translation per se, but can also be influenced by a variety of technical biases or artifacts that are introduced in different steps of a ribo-seq experiment, due to the specific RNase used to digest the unprotected RNAs, the type of antibiotics used to arrest the ribosomes, and the way in which cells are treated with selected antibiotics (e.g., the concentration and timing of antibiotic treatment)^25, 46, 47^. For example, Gerashchenko et al.^48^ suggested that the CHX pre-treatment of cells prior to cell lysis may contribute to the increased RPF density near the start of the CDS. Thus, the elevation of RPF density observed in these regions might not be caused by a decrease in translation elongation. The use of CHX and LTM/Harr may also distort the RPF density near the TISs due to a pause in the subsequent scanning preinitiation complex that is caused by the arrest of the downstream translating ribosomes^46, 47^. This distortion obscures quantitative information on the relative initiation rates. Therefore, it is important to differentiate the effect of translation from that of technical biases or artifacts on the observed signals in ribo-seq data. To reveal the sequence determinants of the RPF read density and help identify potential technical biases or artifacts in ribo-seq data, O’Connor et al.^49^ developed the ribo-seq unit step transformation (RUST) method, a normalization method that was based on the Heaviside step function and that performed better than other methods in the presence of heterogeneous noise. A major difference in the QC metrics offered by RUST and by Ribo-TISH is that the main QC metrics used by RUST for identifying sequencing biases are at the codon level, whereas those used by Ribo-TISH for detecting low-quality reads are at the sub-codon level. We set out to determine whether the QC metrics used by these two methods provide complementary information on the quality of ribo-seq data. Performing QC with Ribo-TISH on a rRibo-seq data set^50^ that was previously shown by RUST^49^ to have excellent quality, we found that this data set showed relatively poor 3-nt periodicity, with *f*_d_ no more than 0.45 at all RPF lengths and the minimum *f*_d_ of 0.37 (Supplementary Fig. 1a). In contrast, we found that the rRibo-seq data generated by Lee et al.^21^ showed excellent 3-nt periodicity, with *f*_d_ no less than 0.69 at all RPF lengths, but showed an apparent sequencing bias based on the RUST analysis (Supplementary Fig. 1b). Therefore, the QC metrics offered by Ribo-TISH and RUST reveal different aspects of ribo-seq data quality and are complementary.

### Modeling the background distribution of TI-seq data

**Fig. 3:**
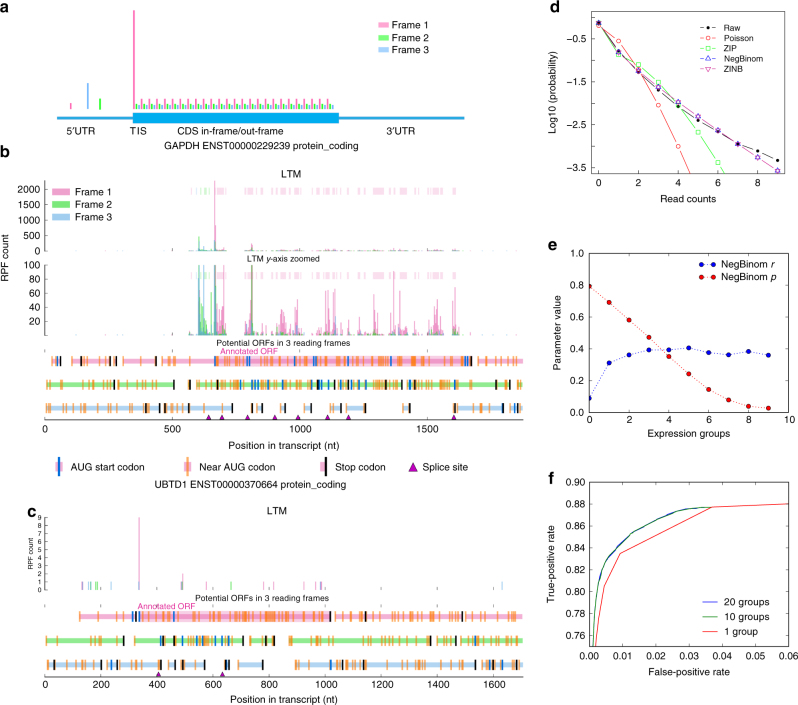
Modeling background distribution of TI-seq data. **a** An illustration of the typical TI-seq RPF count profile across a hypothetical protein-coding transcript. The RPF counts at the first base of CDS in-frame codons (the pink bars inside ORF), excluding AUG or near-cognate start codons, from annotated PCGs, were used to model TI-seq data background. TI-seq RPF count profile for the major isoform of **b** GAPDH and **c** UBTD1. **d** Fitting of different distributions including Poisson, zero-inflated Poisson (ZIP), negative bionomial (NB), and zero-inflated negative binomial (ZINB) to the observed background RPF count distribution. **e** NB distribution parameters (*r* and *p*) estimated from different TI-seq expression groups. **f** The use of different NB background distributions for transcripts/genes with different TI-seq signal density improved TIS identification compared to the use of a single/global NB background distribution

To identify bona fide TISs from TI-seq data, we used the RPF counts at the first base of all CDS in-frame codons (pink bars in CDS region in Fig. 3a), excluding AUG or near-cognate start codons, to model the background distribution. As demonstrated in the examples of *GAPDH* and *UBTD1* (Fig. 3b, c), different transcripts may vary significantly in their level of translation. Using a single distribution to fit the background TI-seq data for all transcripts, regardless of differences in their level of translation, may lead to false positives for highly translated transcripts and false negatives for poorly translated ones. To take into account the different translation levels across transcripts, we divided the transcripts into different groups based on their TI-seq signal density and built different background distributions for each group. We fit the observed background RPF count distribution using four different probability distributions, including Poisson, zero-inflated Poisson (ZIP), negative binomial (NB), and zero-inflated NB (ZINB) distributions. The inclusion of zero-inflated distributions accounts for the potential excess of zero RPF counts^51, 52^ in the non-TISs regions. We performed model selection (Methods) using either the Akaike information criterion (AIC)^53^ or Bayesian information criterion (BIC)^54^. We found that NB and ZINB distributions consistently showed better fit for the background RPF count data across different groups than the other two distributions, with NB distributions being slightly better than ZINB distributions (Fig. 3d, Supplementary Table 1). Consistent with the best fit of the NB distribution to the data, the zero-inflated components estimated in ZINB distributions are <1%. The background distributions for transcripts with different TI-seq signal density were indeed different (Fig. 3e, Methods). Moreover, we found that the use of a different background distribution by grouping the transcripts with similar TI-seq signal density improved the TIS identification based on a receiver-operating characteristic (ROC) analysis using the positive and negative TIS sets that were used in a previous study^12^ and were based on the consensus CDS (CCDS) in Ensembl human gene annotation (Fig. 3f, Methods). The improvement plateaued when the number of groups was over 10 (Fig. 3f).

### Genome-wide identification and differential analysis of TIs

**Fig. 4:**
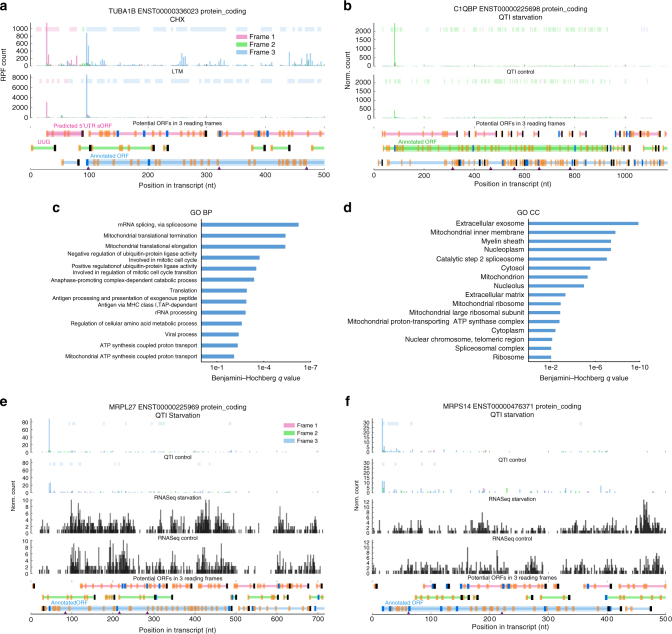
Genome-wide identification and differential analysis of TIs. **a** rRibo-seq and LTM-based TI-seq RPF count profiles in HEK293 cell line for TUBA1B, suggesting a uORF being translated across a different reading frame (pink) from annotated one (skyblue). **b** The normalized QTI-seq RPF count profiles under normal condition and amino-acid deprivation for the longest isoform of *C1QBP*. The top enriched **c** biological processes and **d** cellular components, based on the GO enrichment analysis of the genes with significantly elevated translation initiation efficiency under amino-acid deprivation. The normalized QTI-seq RPF and RNA-seq count profiles under normal condition and amino-acid deprivation for genes encoding mitochondrial ribosomal proteins **e** *MRPL27* and **f** *MRPS14*

After estimating the background distribution of the TI-seq data, Ribo-TISH uses the estimated background distribution to assess the statistical significance of all candidate start codons, including both AUG and near-cognate start codons. For example, an analysis of a published LTM-based TI-seq data set^21^ in human embryonic kidney cells 293 (HEK293) cells revealed that at the *TUBA1B* locus, a uORF may be translated across a reading frame other than the annotated reading frame (Fig. 4a). Ribo-TISH uses the same framework for the TI-seq data generated by different translation inhibitors, including LTM and Harr. To systematically evaluate the performance difference in identifying bona fide TISs between LTM- and Harr-based TI-seq data generated in the same HEK293 cell line^21^, we performed an ROC analysis using the same positive and negative TIS sets as were used in the current study (Methods). We found that the prediction model using LTM-based data showed better performance than that using Harr-based data (Supplementary Fig. 2), with a larger area under the ROC curve (AUC, 0.92 vs. 0.88) and a larger partial AUC (pAUC, 0.79 vs. 0.71) at the false-positive rate (FPR) of 5% (Supplementary Table 2). This result suggests that LTM-based TI-seq may be a better option for genome-wide TIS identification than Harr-based TI-seq. In addition, we found that the AUG or near-cognate TISs identified by Ribo-TISH in a LTM-based TI-seq data set^21^ in HEK293 cells covered over 80% of those that were collected by TISdb, a database for aTI in mammalian cells^33^ (Supplementary Fig. 3a, Fisher’s exact test, *p* < 2.2 × 10^−16^). Furthermore, the CUG and GUG are the top two frequently used non-AUG start codons, and AGG, AAG, and AUA are among the least frequently used non-AUG codons at the predicted TISs, suggesting a robust performance of Ribo-TISH in the presence of potential artifacts in TI-seq data^55^ (Supplementary Fig. 3b).

For the analysis of QTI-seq data (Fig. 4b) to identify differential TISs between two biological conditions, Ribo-TISH uses a stepwise strategy. First, it identifies the TISs under either condition as described in the current and previous section. Second, it takes the union of all TISs identified under two conditions as the candidate TISs for differential analysis. Third, it uses the RPF counts at the candidate TISs between two conditions to perform trimmed mean of *M* values (TMM) normalization^56^. The RNA-seq counts of the corresponding genes are also normalized by TMM. Finally, it uses Fisher’s exact test to assess whether there is a disproportional change of the RPF counts at TISs between two conditions compared with the change in the RNA-seq counts of the corresponding gene (Methods). It also applies a fold change (FC) cutoff to filter out statistically significant differential TISs that only show small FC. When only QTI-seq data are available, Ribo-TISH uses a binomial test to assess the statistical significance of the difference in the normalized RPF counts at TISs between two conditions. Because the observed difference in the QTI-seq signal at the TISs is a composite effect of the difference in TI efficiency and the difference in RNA abundance, QTI-seq data alone would be insufficient for distinguishing whether it is the change in TI efficiency or in RNA abundance that results in the apparent difference in the QTI-seq signal. Therefore, it is necessary to use both QTI-seq and RNA-seq data to detect the change in TI efficiency.

We applied Ribo-TISH to a data set^23^ that contains both QTI-seq and RNA-seq data in HEK293 cells to identify those TISs that show differential TI efficiency between normal and amino-acid deprivation condition. We identified 1145 non-redundant TISs (512 AUG TISs and 633 near-cognate TISs) that showed increased TI efficiency, and 528 non-redundant TISs (382 AUG TISs and 146 near-cognate TISs) that showed decreased TI efficiency upon amino-acid deprivation (FDR ≤ 0.05, |log_2_FC| ≥ log_2_1.5). Among the TISs with upregulated or downregulated TI efficiency, the dominant classes are the TISs of uORFs and the annotated ORFs, the combination of which consists 79% of all upregulated TISs (Supplementary Fig. 4a) and 83% of all downregulated TISs (Supplementary Fig. 4b). We further performed Gene Ontology (GO)-based functional enrichment analysis^57^ using DAVID (http://david.ncifcrf.gov/) to identify the biological processes that are enriched for PCGs with elevated TI efficiency at annotated TISs upon amino-acid deprivation (Methods). The top enriched biological processes include many fundamental processes, such as mRNA splicing, ubiquitin-protein ligase activity, cell cycle, and translation (Fig. 4c). Interestingly, mitochondrial translation elongation and termination are among the top three enriched biological processes (Fig. 4c). The GO-based enrichment analysis of cellular components also showed that the PCGs with elevated TI efficiency at annotated TISs, upon amino-acid deprivation, were enriched in the mitochondrial compartment (Fig. 4d). Some of the large (Fig. 4e) and small units (Fig. 4f) of the mitochondrial ribosome showed significantly increased TI efficiency during amino-acid deprivation. These results suggest an important role of elevated mitochondrial translation for mammalian cells to cope with the stress of amino-acid deprivation. Consistent with our computational finding, a recent study^58^ using ^35^S-methionine pulse-chase labeling of nascent mitochondrial polypeptides showed that amino-acid starvation indeed enhanced mitochondrial protein synthesis as well as increased mitochondrial respiration and membrane potential.

### Ribo-TISH outperformed existing methods in predicting ORFs

**Fig. 5:**
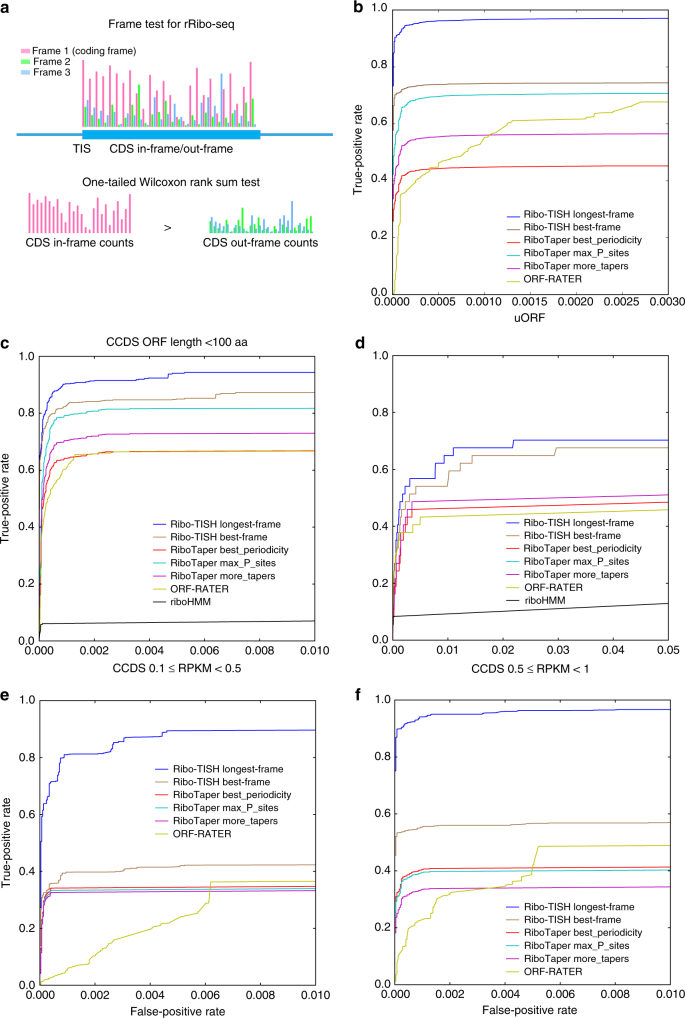
Evaluating the performance of different methods in ORF prediction. **a** An illustration of how the frame test was performed to predict ORFs from rRibo-seq data by Ribo-TISH. **b** ROC curves across six strategies of ORF predictions implemented in Ribo-TISH, RiboTaper, and ORF-RATER. An RPKM value of 1 was used as a cutoff to define actively translating genes for positive and negative sets. **c** The short ORFs (<100 aa) of CCDS in Ensembl or **d** the experimentally validated uORFs curated by uORFdb were used as a positive set (RPKM ≥ 1) for the ROC analyses across 7 strategies implemented in Ribo-TISH, RiboTaper, ORF-RATER, and riboHMM. ROC curves across six strategies implemented in Ribo-TISH, RiboTaper, and ORF-RATER when the annotated ORFs of CCDS in Ensembl, with RPKM **e** between 0.1 and 0.5 or **f** between 0.5 and 1, are used as a positive set, respectively

In addition to genome-wide identification and differential analysis of TIs from TI-seq data, Ribo-TISH allows for predicting putative ORFs from CHX-based rRibo-seq data. Those actively translated ORFs are expected to have significantly more RPF counts from the bona fide reading frame than from the alternative reading frames, also known as 3-nt periodicity. Ribo-TISH uses a frame test based on the non-parametric Wilcoxon rank-sum test (Methods) to quantitatively assess the difference in read counts at individual CDS nucleotide positions between the candidate and alternative reading frames to predict the translated reading frame (Fig. 5a). To evaluate the performance of Ribo-TISH in predicting ORFs from rRibo-seq data, we performed an ROC analysis for Ribo-TISH and four other published methods: RiboTaper^40^; ORF-RATER^41^; riboHMM^42^; and RibORF^12^ using a published rRibo-seq data set^21^ (Methods). Like Ribo-TISH, RiboTaper, ORF-RATER, and riboHMM can predict ORFs from rRibo-seq data without user-specified ORF candidates. RiboTaper was built upon the multi-taper method developed in the signal-processing field. RiboTaper and Ribo-TISH use unsupervised methods that do not rely on prior knowledge of the ORF annotation and allow for de novo prediction of ORFs from rRibo-seq data. In contrast, ORF-RATER and riboHMM utilize supervised approaches that require training on the rRibo-seq data of the annotated ORFs. ORF-RATER is based on the linear regression method and riboHMM uses a hidden markov model. Different from the other methods, RibORF is a candidate-based method that requires the user to provide a list of candidate ORFs for prediction and it utilizes a supervised approach built on support vector machines. In addition to these fundamental algorithmic differences, Ribo-TISH supports more functionality than the other tools (Supplementary Table 3 **)**. In particular, only Ribo-TISH provides the functionality to assess whether a given RPF read is compatible with the splice junctions of the annotated isoforms (Supplementary Fig. 5).

We first compared the performances of Ribo-TISH, RiboTaper, ORF-RATER, and riboHMM in the prediction of ORFs from rRibo-seq data without user-specified ORF candidates. In contrast to Ribo-TISH, which allows for de novo prediction of ORFs with AUG or near-cognate start codons, RiboTaper does not support the prediction of ORFs with non-AUG start codons, and ORF-RATER is too computationally demanding to predict ORFs with non-AUG start codons (see the comparison of computational efficiency). To make a fair comparison of the different methods, we focused on the annotated ORFs with the AUG start codon for the ROC analysis. In total, we evaluated seven different strategies (Methods) of ORF predictions, including two implemented in Ribo-TISH, three in RiboTaper, one in ORF-RATER, and one in riboHMM. Using the positive and negative ORF sets based on the CCDS in Ensembl annotation (Methods) and reads per kilobase of transcript per million mapped reads (RPKM) of 1 as the cutoff for defining the actively translating genes, we found that the longest-frame strategy implemented in Ribo-TISH showed the best predictive performance among all the strategies of ORF prediction (Fig. 5b), with both AUC and pAUC at 1% FPR being > 0.96. The strategy with the second best performance was the best-frame strategy implemented in Ribo-TISH, with an AUC of 0.87 and a pAUC (FPR = 0.01) of 0.74. The best or only strategy implemented in RiboTaper (max_P_sites) and ORF-RATER resulted in similar performances to each other, with an AUC of 0.85 vs. 0.83, and a pAUC (FPR = 0.01) of 0.70 vs. 0.64. Because the ORFs predicted by riboHMM in protein-coding transcripts were dominantly uORFs (Supplementary Fig. 6a) and short ORFs (Supplementary Fig. 6b), we only evaluated its performance in predicting canonical ORFs shorter than 100 amino acids (aa) and uORFs (see later performance comparison in this section). Better predictive performance was consistently observed for Ribo-TISH when a different RPKM threshold of 10 was used to define the actively translating genes (Supplementary Fig. 7 and Supplementary Table 4). We further compared the performance of Ribo-TISH and RibORF in the candidate-based prediction of ORFs. We found that Ribo-TISH showed superior performance compared to RibORF (Supplementary Fig. 8a and Supplementary Table 5), with both AUC and pAUC (FPR = 0.01) > 0.98. In contrast, although RibORF had a total AUC similar to that of Ribo-TISH, its pAUC was about 0.87. Better predictive performance of Ribo-TISH was again consistently observed when a different threshold was used to define the actively translated genes (Supplementary Fig. 8b and Supplementary Table 5).

Because the longest-frame strategy implemented in Ribo-TISH showed better performance than all the other methods for predicting ORFs with AUG start codons, an important issue is whether its superior performance is simply due to certain bias toward longer ORFs. To address this issue, we performed an ROC analysis using as a positive set the annotated ORFs of the CCDS in Ensembl that are shorter than 100 aa. For this group of ORFs, the longest-frame strategy remained the top performer, followed by the best-frame strategy (Fig. 5c). In addition to their superior performances in predicting canonical ORFs, the longest-frame strategy and the best-frame strategy showed better performances than the other methods in predicting the experimentally validated uORFs from uORFdb, a uORF database based on literature curation^59^ (Fig. 5d). Therefore, it is very unlikely that the better performance of the longest-frame strategy is simply due to bias toward longer ORFs. Both the longest-frame strategy and the best-frame strategy are based on the same frame test. The only difference between these two strategies is that when there are multiple in-frame candidate ORFs that share the same stop codon, the best-frame strategy selects the ORF that shows the best *p*-value of the frame test, whereas the longest-frame strategy selects one with the most upstream TIS as long as the frame test result is significant. Because the RPF reads are nonuniformly distributed across the CDS, for in-frame candidate ORFs that share the same stop codon, the ORF selected by the longest-frame strategy may differ from the one selected by the best-frame strategy. The observed better performance of the longest-frame strategy might reflect the underlying biology of the canonical translation: among multiple in-frame AUG-initiating ORFs that share the same stop codon and show good 3-nt periodicity in rRibo-seq data, it is more likely that the first encountered AUG will be utilized. However, for predicting ORFs with near-cognate start codons, the longest-frame strategy may not be a good strategy because the most upstream candidate near-cognate start codon may not have superior initiation strength.

One of the challenges in predicting ORFs from rRibo-seq data is to predict lowly expressed ones. To evaluate the performance of different methods for predicting lowly expressed ORFs, we stratified the annotated ORFs based on their expression level measured from rRibo-seq data and performed an ROC analysis on ORFs with relatively low expression. We found that the performance of Ribo-TISH was consistently superior to that of other methods in predicting lowly expressed ORFs (Fig. 5e, f). The one-tailed and non-parametric nature of the frame test makes Ribo-TISH more robust and less sensitive to abnormal RPF counts at individual nucleotide positions due to background noise originating from sequencing biases or contaminations of non-ribosome-bound RNA and regulatory RNA in the ribosomal complex. This is important especially when the total RPF count within CDS is relatively low and/or the RPF reads are sparse and nonuniformly distributed across the whole ORF, because if there are a small fraction of nucleotide positions with abnormal RPF counts, the RPF counts from the other positions within the same CDS would still provide sufficient information to capture the RPF enrichment in the truly translated frame.

The rRibo-seq data set that we used for the performance evaluation showed excellent 3-nt periodicity (*f*_d_ ≥ 0.69) at all RPF lengths (Supplementary Fig. 1b). As a result, no RPF reads were filtered out in the analysis. In practice, it is more likely that a rRibo-seq data set may contain a fraction of RPFs with low quality. To assess the effect of removing the RPFs with poor 3-nt periodicity on ORF prediction for different methods, we chose the ribo-seq data set from Fig. 2c that has a mixture of the RPF reads of high quality (~71.5%) and those of low quality (~28.5%) based on the QC metrics provided by Ribo-TISH. We compared the performance of the different methods on the data with (filtered) or without (unfiltered) removing the RPFs with the lengths, at which the *f*_d_ is < 0.5. Both the longest-frame and the best-frame strategy from Ribo-TISH showed improved performances on the filtered data over those on the unfiltered data (Supplementary Fig. 9a, b). Similarly, all three strategies from RiboTaper (Supplementary Fig. 9c–e) showed improved performances on the filtered data compared to those on the unfiltered data. In contrast, the ORF-RATER showed almost the same performance on filtered and unfiltered data (Supplementary Fig. 9f). This result might be because ORF-RATER does not explicitly rely on the 3-nt periodicity pattern in ribo-seq data for ORF prediction. Although this feature might make ORF-RATER more robust when only a fraction of RPFs have poor 3-nt periodicity, a potential issue is that ORF-RATER may predict ORFs on a data set with overall poor 3-nt periodicity. Consistently, we found that ORF-RATER predicted (with default thresholds) ~600 annotated AUG-initiated ORFs of the CCDS in Ensembl on an RNA-seq data set^60^ (GSM1306496) in HEK293 cells, which are all supposed to be false-positive predictions; whereas, Ribo-TISH predicted zero and RiboTaper only predicted 11 ORFs of the CCDS in Ensembl with their default thresholds on the same RNA-seq data.

Aside from prediction accuracy, we compared the computational efficiency of the different methods (Methods) using a published rRibo-seq data set of 25 M mapped RPF reads^21^ and an RNA-seq data set (only required by RiboTaper) of 34 M mapped reads^60^ (see Data Sources in Methods). Because RibORF and riboHMM do not support parallel computing, we only included Ribo-TISH, RiboTaper, and ORF-RATER for this comparison. Given that RiboTaper does not support the prediction of ORFs with non-AUG start codons, the prediction was restricted to ORFs with AUG start codons. We found that Ribo-TISH was about 35 times faster than RiboTaper and was about 8 times faster than ORF-RATER in predicting ORFs with AUG start codons. In addition, Ribo-TISH used around 1/41 and 1/22 of the memory that RiboTaper and ORF-RATER required, and used much less hard disk space for intermediate files (Supplementary Table 6). The high CPU memory demand of RiboTaper and ORF-RATER may create challenges for an average user who has no access to high-performance computing resources and has to use these tools on a desktop or laptop computer. In addition, Ribo-TISH outperformed ORF-RATER in computational efficiency for the prediction of ORFs with NUG (N = A, U, C, and G) start codons (Supplementary Table 7). ORF-RATER required more than 180 GB of CPU memory and encountered an issue of memory overflow.

### Experimental validation of new smORFs predicted by Ribo-TISH

As a real application of Ribo-TISH for ORF prediction, we applied Ribo-TISH to a published data set from the HEK293 cell line with both LTM-based TI-seq data and rRibo-seq data^21^. We focused on predicting novel ORFs that are completely different from the annotated ORFs (i.e., not the truncated/extended isoforms of the known ORFs). By statistically integrating the TIS prediction from TI-seq data and the ORF prediction from rRibo-seq data (Methods), we predicted 5032 novel ORFs, including 4268 (85%) ORFs from 5′UTRs (uORFs), 42 (1%) ORFs from 3′UTRs (dORFs), 176 (3%) ORFs that are internal to and out-of-frame with the known ORFs (internal), and 546 (11%) ORFs from lncRNAs (Supplementary Fig. 10a). Interestingly, we found that the start codon usage in uORFs is distinct from that for the other classes of ORFs (Supplementary Fig. 10b). This finding was consistent with previous studies^21, 22^. Only about 30% of the predicted uORFs initiate at the AUG start codon, whereas more than 50% of the other classes of ORFs initiate at the AUG start codon. The most frequent near-cognate start codon in uORFs, dORFs, and the ORFs from lncRNAs is CUG. In contrast, ACG is the most frequent near-cognate start codon in internal ORFs. In addition to the difference in the start codon usage, the predicted ORFs from different classes exhibit distinct length distributions (Supplementary Fig. 10c). The uORFs and internal ORFs have a median length around 30 aa, whereas the dORFs and the ORFs from lncRNAs have a median length of around or over 50 aa.

**Fig. 6:**
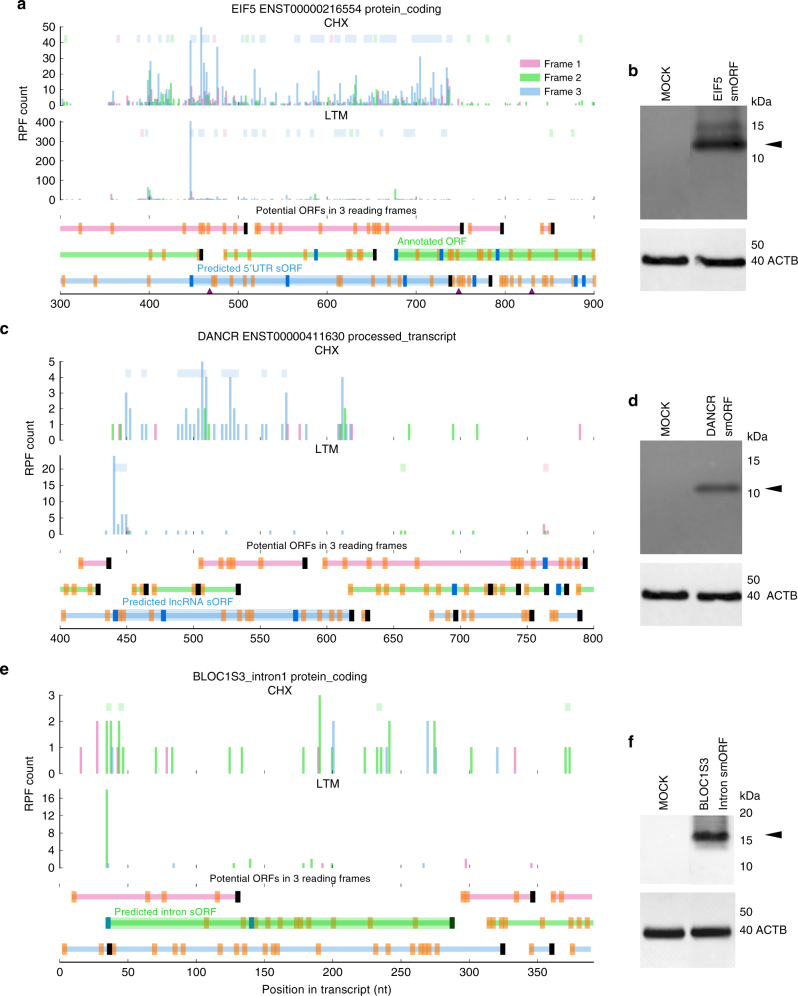
Experimental validations of the computationally predicted smORFs. **a** rRibo-seq and LTM-based TI-seq RPF count profiles in HEK293 cell line for a predicted uORF in 5′UTR of *EIF5*. **b** FLAG-tagged uORF within the context of the host mRNA was ectopically expressed and translation of the predicted polypeptide was detected by western blot with an anti-FLAG antibody. β-actin protein was used as internal control for western blot analysis. **c** rRibo-seq and LTM-based TI-seq RPF count profiles for a predicted ORF encoded by lncRNA *DANCR*. **d** FLAG-tagged smORF within the context of host lncRNA was ectopically expressed and translation of the predicted polypeptide was detected by western blot with an anti-FLAG antibody. **e** rRibo-seq and LTM-based TI-seq RPF count profiles for a predicted ORF encoded by an intron of *BLOC1S3*. **f** FLAG-tagged intronic smORF within the context of the host mRNA was ectopically expressed and translation of predicted polypeptide was detected by western blot with an anti-FLAG antibody

To experimentally validate the predicted novel ORFs, we focused on smORFs with lengths between 50 and 100 aa, and with the canonical AUG start codon, which consist of 248 uORFs, 99 lncRNA-encoded, and 3 intron-encoded ORFs (Supplementary Table 8). For smORFs encoded by lncRNAs, we required both the smORFs and the corresponding lncRNAs to be conserved between humans and other primates^61–63^ (Methods and Supplementary Table 8). We selected one top smORF candidate in each category from 5′UTRs, lncRNAs, and the introns of PCGs for validation (Supplementary Table 9). We also generated a rRibo-seq (Methods) data set in HEK293 cells (GSE94460) to confirm that the top smORF candidates were likely to be translated based on this independent data set. We tested whether the predicted smORF-encoding transcripts were competent to produce a polypeptide by first ectopically expressing the corresponding host mRNAs or lncRNAs that encode the smORFs with a 3′ end addition of FLAG epitope tags and then detecting the translated polypeptide by western blot analysis with an anti-FLAG antibody (Methods, Supplementary Table 10). We first confirmed the polypeptide produced by the top uORF candidate from the 5′UTR of *EIF5* (Fig. 6a, b). For the smORF encoded by lncRNAs, we chose the second best candidate smORF from the lncRNA *DANCR* for experimental validation, because the top candidate lncRNA *GAS5* is known to undergo nonsense-mediated decay and encode smORFs^64, 65^. Consistent with our prediction (Fig. 6c), a polypeptide encoded by *DANCR* was detected with the expected size from the western blot analysis (Fig. 6d). For the top intronic smORF candidate within an intron of *BLOC1S3* (Fig. 6e), the detected size (~15 kDa) of the polypeptide (Fig. 6f) was different from the computational prediction (~12 kDa). To confirm the true identity of this polypeptide, we performed immunoprecipitation coupled with mass spectrometry analysis. The detected sequences of the polypeptide indeed corresponded to the predicted smORF sequences (Supplementary Table 11, Supplementary Figs. 11–15), but did not correspond to any other protein in the human proteome. Interestingly, the smORF-encoding intron of *BLOC1S3* is in the 5′UTR region. Introns in 5′UTR can play an important regulatory role in the nuclear export of the mRNAs through their nucleotide sequences^66, 67^. Our finding of the smORF-encoding intron in the 5′UTR region suggests that the 5′UTR introns might influence gene expression through a coding-dependent mechanism, which awaits further studies.

## Discussion

Translational control is critical for gene regulation during many developmental, physiological, and pathophysiological processes, and occurs principally at the initiation stage. Recent studies using TI-seq/QTI-seq and/or rRibo-seq techniques have revealed a notably complex translational landscape in metazoans, with hundreds of novel smORFs outside the known PCGs and with many mouse/human genes that have aTI isoforms. Evidence is mounting that some of these smORFs/aTI isoforms can serve important biological functions. Despite the broad applicability and wide adoption of TI-seq/QTI-seq and rRibo-seq techniques, the lack of computational tools that facilitate efficiently and comprehensively decoding the translational landscape from different types of ribo-seq experiments presents a major challenge to unleashing the full power of ribo-seq data.

Ribo-TISH is a comprehensive informatic solution to this challenge. It enables both low-level and high-level analysis of TI-seq/QTI-seq data, starting from QC of the aligned sequencing data to identifying and quantitatively comparing genome-wide TIs under different conditions. In addition, it allows for predicting actively translated ORFs from CHX-based rRibo-seq data. Ribo-TISH outperformed several other published methods for ORF prediction from rRbio-seq data in both computational efficiency and prediction accuracy. In particular, Ribo-TISH improved the prediction accuracy for genes with low expression and enabled computationally efficient de novo prediction of ORFs with near-cognate start codons.

Many technical biases or artifacts originating from different experimental sources have been observed in ribo-seq data^46, 47^. Therefore, the QC of ribo-seq data is important for identifying the potential biases that may affect the biological conclusions that are drawn. Using the appropriate QC can also improve experimental design, protocol selection, and downstream data analyses. We demonstrated that removing the RPFs with low quality from rRibo-seq data based on the QC metrics provided by Ribo-TISH improved the performance in ORF prediction for most computational strategies that we evaluated. Moreover, the QC metrics provided by Ribo-TISH offer information about the data that differs from that provided by an alternative method, RUST. In practice, it is advisable to use several methods for QC of ribo-seq data to have a comprehensive view of the data quality. Although the LTM- or Harr-based TI-seq experiments are powerful for mapping the TISs, the signals at the TISs from these experiments do not necessarily reflect the true TI rates and cannot be used for quantitative comparisons between conditions^23, 46, 47^. Therefore, a QTI-seq^23^ experiment is necessary for any study that aims to identify differential TIs between conditions. Ribo-TISH provides both the functionality of analyzing QIT-seq data alone to identify differential TISs with apparent change in the QTI-seq signal, and the functionality of jointly analyzing QTI-seq and RNA-seq data to identify differential TISs due to the change in TI efficiency between conditions. For the former functionality, Ribo-TISH can analyze the data with a single replicate on its own, as well as analyze the data from replicates with the aid of other tools such as edgeR^68^ and DESeq2^69^. For the latter functionality, Ribo-TISH currently can only analyze the data with a single replicate. It is important to further develop the method to enable a joint analysis of QIT-seq and RNA-seq data from replicates.

Recent studies^70, 71^ have shown that different RNA transcript isoforms can be subject to differential translational control that expands a large dynamic range, suggesting the importance of characterizing transcript-isoform-specific translational regulation. Ribo-TISH can distinguish whether RPF reads are compatible with the splicing patterns of the given transcript isoforms, whereas this feature was not supported by any of the other tools (Supplementary Table 3 and Supplementary Fig. 5). In its current implementation, Ribo-TISH treats the transcript isoforms from the same gene independently without jointly modeling the RPF sequencing reads across isoforms. Therefore, it does not quantify the TI at the level of the individual isoform. Similar to the other published methods, Ribo-TISH does not provide functionality for inferring isoform-level ribosome occupancy to predict ORF translation. In the future, it will be important to develop statistical models that enable joint analysis of the RPF reads across different transcript isoforms of the same gene to infer the isoform-specific TI or ORF translation, as was done for isoform quantification from RNA-seq data in previous studies^72–77^.

When applied to published TI-seq/QTI-seq and rRibo-seq data sets, Ribo-TISH uncovered a novel signature of elevated mitochondrial translation during amino-acid deprivation in HEK293 cells, suggesting that the elevated mitochondrial translation may be an important and integrated component of the amino-acid deprivation-induced stress response process. Importantly, this computational finding was experimentally validated by an independent study, where the authors showed that amino-acid starvation enhanced mitochondrial protein synthesis by using ^35^S-methionine pulse-chase labeling of nascent mitochondrial polypeptides^58^. Ribo-TISH also predicted many novel ORFs, including one encoded by the lncRNA *DANCR* and one encoded by the 5′UTR of *EIF5*, both of which were experientially confirmed. Interestingly, it revealed a novel ORF within a previously annotated intron in the 5′UTR of the PCG *BLOC1S3*, which was experimentally validated. Approximately 35% of human 5′UTRs are annotated as harboring introns^66^. Genes with regulatory functions are enriched for introns in the 5′UTRs, whereras the 5′UTR introns are significantly depleted in genes that encode proteins targeted to the mitochondria or edoplasmic reticulum^78^. Introns in 5′UTRs can influence gene expression through different mechanisms (e.g., dictating the mechanisms of mRNA export) from those used by introns in CDS^66, 67^. Our finding of the first smORF-encoding intron in the 5′UTR region suggests that some of these previously annotated 5′UTR introns might influence gene expression through a coding-dependent mechanism.

In summary, Ribo-TISH is a computationally efficient toolkit for decoding the translational landscape from both TI-seq/QTI-seq and rRibo-seq data. It promises to benefit the broad research community in studies of the function and mechanisms of translational regulation under different contexts.

## Methods

### Ribosome profiling and library preparation

Sample preparation for ribosome profiling was conducted according to the manufacturer’s specifications for the TruSeq Ribo Profile (Mammalian) Library Prep Kit (Illumina). Briefly, HEK293 cells were treated with CHX (Sigma-Aldrich, final concentration 0.1 mg/ml) for 1 min. In-dish cell lysis was performed using mammalian lysis buffer (including CHX at a concentration of 0.1 mg/ml). Then 600 μl of lysate were taken and 15 μl of RNase I (100 U/μl, Thermo Fisher Scientific) were added and the mixtures were incubated for 45 min at room temperature, followed by adding 15 μl SUPERaseIn RNase inhibitor (Ambion, Thermo Fisher Scientific) to stop the reaction. Ribosome recovery was performed by illustra MicroSpin S-400 HR Columns (GE Healthcare) and the RPFs were purified by RNA Clean & Concentrator (Zymo Research). Ribosomal RNAs were depleted using Ribo-Zero Magnetic Gold Kit (Human/Mouse/Rat, Illumina). RPFs without ribosomal RNA were run on a 15% urea denaturing-PAGE gel, and the gel slices corresponding to 28–30 nts were excised. The RPF RNAs were eluted and precipitated followed by library construction according to the manufacturer’s protocol.

### Cloning

The fragments that concatenate the 5′-upstream sequences, the CDS of putative smORFs (without stop codon), and a 3′-3xFLAG-epitope along with a stop codon were generated by synthesizing gBlocks gene fragments (IDT) followed by polymerase chain reaction. The products were cloned into the multiple cloning sites XbaI and BamHI of pcDNA3.1(-) under a cytomegalovirus promoter. The primer sequences and the sequences of the synthesized gBlock are listed in Supplementary Table 10.

### Cell culture and transient transfection

HEK293 cells (gifts from Dr. George A. Calin’s lab) were grown in high-glucose Dulbecco’s modified Eagle’s medium supplemented with 10% fetal bovine serum, penicillin, and streptomycin at 37 °C under an atmosphere of 5% CO_2_ and plated in six-well plates 24 h before transfection. SmORF-3xFLAG constructs and a mock negative control with start codon-removed 3xFLAG sequence in the same plasmid backbone were transfected into the cells with Lipofectamine 3000 Reagent (Invitrogen, Thermo Fisher Scientific). The identity of HEK293 cells was authenticated by short tandem repeat fingerprinting at the Characterized Cell Line Core Facility of UT MD Anderson Cancer Center. Mycoplasma contamination of HEK293 cells was tested using MycoAlert PLUS Mycoplasma Detection Kit (Lonza, LT07-703) and the result was negative.

### Western blot

Forty-eight hours post transfection, the HEK293 cells were lysed using CelLytic M lysis reagent (Sigma-Aldrich). Clarified cell lysates (30 μl) were mixed with 2× Tricine SDS Sample Buffer (Novex, Thermo Fisher Scientific) and run on 10–20% Tricine Protein Gels (Novex, Thermo Fisher Scientific) in Tricine SDS Running Buffer (Novex, Thermo Fisher Scientific) at 125 V for 90 min. Proteins were transferred to polyvinylidene fluoride membrane (0.2 μm, Bio-Rad) at 100 mA for 2 h in Tris-Gly Transfer Buffer (Novex, Thermo Fisher Scientific) supplement with methanol (Sigma-Aldrich). Immunoblots were incubated with primary monoclonal anti-FLAG M2 antibody (1:1000, F1804-200UG, Sigma-Aldrich) and anti-β-actin (1:10,000, AM4302, Ambion, Thermo Fisher Scientific) overnight at 4 °C and then secondary anti-mouse IgG and horseradish peroxidase-linked antibody (1:5000, Cell Signaling) at room temperature for 2 h. Immunoblots were developed with Western ECL (Clarity, Bio-Rad). Full, uncropped versions of all blot images are provided in Supplementary Fig. 16.

### Immunoprecipitation and mass spectrometry sample preparation

HEK293 cells in 10 cm^2^ plates with 80% confluency were transfected with 10 µg smORF-3xFLAG constructs and 10 µg mock vector control 48 h prior to immunoprecipitation. Transfected cells were harvested in CelLytic M lysis buffer coupled with protease inhibitor cocktail (Sigma-Aldrich). Extracts were incubated at 4 °C overnight with 50 µl anti-FLAG M2 affinity gel (Sigma-Aldrich). The resulting immune complexes were washed, and the FLAG-tagged polypeptides were eluted by a competition with 3xFLAG peptide (Sigma-Aldrich) in wash buffer followed by western blot analysis with monoclonal anti-FLAG M2 antibody (1:1000, F1804-200UG, Sigma-Aldrich). After immunoprecipitation, proteins were separated on a pre-cast Tricine SDS-PAGE gel (Bio-Rad) and stained with GelCode Blue Stain reagent (Thermo Fisher Scientific). Gel slices were excised and digested with trypsin (Promega) overnight at 37 °C. Digested peptides were analyzed by liquid chromatography-tandem mass spectrometry (LC-MS/MS) using an Ultimate 3000 system (Dionex) coupled to an Orbitrap Elite mass spectrometer (Thermo Fisher Scientific). Data were interrogated with smORF sequence by the Mascot Software (version 2.5.1) through Proteome Discoverer (Thermo Fisher Scientific)

### RPF reads alignment

The RPF reads were trimmed and the low-quality reads were filtered by Sickle (http://github.com/ucdavis-bioinformatics/sickle). The RPF reads after filtering were mapped to human rRNA sequences using bowtie and allowing for two mismatches. The reads that were not mapped to human rRNA sequences were then mapped to human genome (GRCh38) with Ensembl gene annotation release 83 using STAR^79^. The alignment was performed with the following parameters: “–outSAMattributes All–outFilterMismatchNmax 2–alignEndsType EndToEnd–outFilterIntronMotifs RemoveNoncanonicalUnannotated–alignIntronMax 20000–outMultimapperOrder Random–outSAMmultNmax 1”.

### Compatibility of RPF reads with transcript structur**e**

For an RPF read that overlaps with a transcript, the exon-intron structure of the transcript within their overlapping genomic region was extracted and evaluated. Only the RPF read that is consistent with the exon-intron structure of a transcript will be assigned to this transcript (see the detailed examples in Supplementary Fig. 5).

### Quality control of ribo-seq data

Quality control was performed using all the uniquely mapped RPF reads in the annotated ORFs of the CCDS in Ensembl human gene annotation version 83. The longest ORF was used for each gene. RPFs were grouped by their lengths and whether the base of their 5′ end matches the genome. Each aligned RPF read was represented by its 5′ end before estimation of the P-site offset.

The RPF count between the 15 bp upstream of the first base of the start codon and the 12 bp upstream of the first base of the stop codon were used to calculate the RPF count distributions across three reading frames. The fraction of the RPF counts in the dominant frame (*f*_d_) was calculated as the ratio between the maximum RPF count among all three reading frames and the sum of the RPF counts from all reading frames. For rRibo-seq data, if the *f*_d_ of the RPF reads of a given length was lower than a user-definable threshold (default 0.5), this group of RPF reads was considered to be of low quality and was discarded in downstream analysis.

The metagene RPF count profile near the start/stop codon was constructed by summing the RPF count between −40 and +20 bp of the first base of the start/stop codon across all annotated PCGs. The P-site offset was estimated based on the distribution of the 5′ end of the metagene RPF counts near the annotated start codons. All estimated P-site offsets were saved in a python script file. RPFs were represented by their P-site positions in downstream analysis. After P-site offset correction, the ratio (*f*_t_) between the RPF counts at the annotated TISs and the sum of the RPF counts near the annotated TISs (from −1 to +1 relative to the annotated TISs) was calculated at different RPF lengths for TI-seq data. Similar to the case of rRibo-seq data, Ribo-TISH keeps only the RPF lengths, at which *f*_t_ is above a user-definable threshold (default 0.5) for downstream analysis. The CDS metagene profile was constructed using the RPF counts in the region between 15 bp upstream of the first base of the start codon and 12 bp upstream of the first base of the stop codon. The CDS metagene profile was constructed for three reading frames, respectively. For each frame, the CDS region was divided into 20 bins and the average RPF count across all annotated PCGs was calculated for each bin. For TI-seq data, a TIS enrichment score was also calculated as the RPF count at TIS divided by the mean RPF count across the whole CDS in the corresponding reading frame.

### Data sources

For the demonstration of how Ribo-TISH performs QC of TI-seq and rRibo-seq data, an LTM-based TI-seq data (SRR618772 and SRR618773) in human HEK293 cells^21^, a Harr-based TI-seq data (SRR315607) in mouse embryonic cells^22^, and an rRibo-seq data (SRR970588) in human HeLa cells were used^45^. For the other analyses, the LTM-based TI-seq data (SRR618772 and SRR618773), the rRibo-seq data (SRR618770 and SRR618771), and the Harr-based TI-seq data (SRR964946) from a published data set^21^ in human HEK293 cells were used. The QTI-seq data under normal condition (SRR1630828) and amino-acid deprivation (SRR1630828) and the corresponding RNA-seq data (SRR1630838 and SRR1630840) were obtained from another data set^23^. RNA-seq data^60^ (GSM1306496) was used as the input for RiboTaper. Ensembl human gene annotation version 83 was used as transcript annotation.

### Model selection for TI-seq background distribution

Four discrete probability distributions, including Poisson, ZIP, NB, and ZINB, were tested for modeling the background distribution of TI-seq read counts. The transcripts were divided into 10 groups based on the TIS read density of the transcript and each group has the same number of total TI-seq read counts. The parameters of the four models were determined by fitting the observed distribution of the RPF counts at the first base of all the codons that are in frame with the known ORFs, excluding AUG or potential near-cognate start codons. Model selection was performed using AIC^53^ and BIC^54^. The distribution fitting and AIC/BIC calculations were performed using the function “fitdist” implemented in the R package fitdistrplus.

### TIS and ORF prediction

NB models were used to model the background distribution of TI-seq/QTI-seq data for testing TI-seq/QTI-seq signal enrichment at the candidate TISs. The parameters of the background NB model were estimated by fitting the observed distribution of the RPF count at the first base of all the codons that are in frame with the known ORFs, excluding AUG or potential near-cognate start codons. For Harr-based TI-seq data, the first 15 codons starting from TIS were excluded. Ribo-TISH divided the transcripts into 10 groups by default based on the TIS read density of the transcript, and each group has the same number of total TI-seq read counts. After NB parameters were estimated for these expression groups, each transcript was assigned to one of the groups, and one-tailed NB test was performed to assess the statistical significance of all candidate start codons. For predicting actively translated ORFs from rRibo-seq data, each nucleotide position in the CDS was divided into the positions from the candidate reading frame and those from the alternative reading frames. The number of RPF counts at each position from the candidate reading frame makes up the first group and that at each position from the alternative reading frames makes up the second group. A frame test was performed, by using a one-tailed Wilcoxon rank-sum test, to assess whether the RPF counts from the first group are generally higher than those from the second group.

### Differential analysis of TISs

After the TISs under the conditions of interest were identified, a TMM^56^ normalization factor was calculated based on the RPF counts at the union of significant TISs under these conditions. TMM normalization is used by edgeR^68^ for the normalization of RNA-seq data. Briefly, *M* values (log ratio) and *A* values (mean average) were calculated for these TISs. The TISs with the highest and lowest 30% of *M* values were trimmed, and TISs with highest and lowest 5% of *A* values were also trimmed. The weighted mean of the remaining *M* values was calculated and used as the normalization factor. The binomial test was used to assess the statistical significance of the difference in normalized RPF counts between the TISs of the two conditions when only a single replicate of QTI-seq data are available and no RNA-seq is available. When QTI-seq data have replications, Ribo-TISH exports TIS count table and provides R scripts to call differential TISs using edgeR^68^ or DESeq2^69^. To identify TISs with up or downregulated TI efficiency by jointly analyzing QTI-seq and RNA-seq data, a Fisher’s exact test was performed on the normalized TIS and RNA-seq counts to assess whether there is a disproportional change of the RPF counts at TISs between two conditions compared with the change in the RNA-seq counts of the corresponding gene. A FC cutoff of 1.5 was further applied to filter out TISs that only show a small FC.

### Positive and negative sets for performance evaluation

For a general evaluation of the performance of different methods, the annotated TISs and ORFs of the CCDS in Ensembl human gene annotation version 83 were used as the positive set. The CCDS protein set is a core set of protein-coding regions in human and mouse, which are consistently annotated across databases and of high quality. The CCDS is a collaborative effort across several annotation databases (http://www.ncbi.nlm.nih.gov/projects/CCDS/CcdsBrowse.cgi). The out-of-frame ORFs inside the CCDS and from the predicted ORFs within short noncoding transcripts were used as the negative set. For the comparison of performance in predicting short ORFs or uORFs, the CCDS ORFs that are shorter than 100 aa or experimentally validated uORFs from uORFdb were used as the positive set, respectively.

### Performance of different methods in predicting ORFs

There can be multiple candidate in-frame ORFs that share the same stop codon in de novo prediction of ORFs from rRibo-seq data. The ability of finding the correct TIS for each annotated ORF using rRibo-seq data is part of our evaluation. Ribo-TISH provides two strategies named “longest frame” and “best frame” for de novo prediction of ORFs based on rRibo-seq data. Both strategies use the one-tailed Wilcoxon sum test to quantitatively assess the 3-nt periodicity. They differ when there are multiple candidate in-frame ORFs that share the same stop codon: the best-frame strategy selects the ORF that shows the best *p*-value of the frame test, whereas the longest-frame strategy selects the one with the most upstream TIS as long as the frame test result is significant. RiboTaper provides three strategies including best_periodicity, max_P_sites, and more_tapers, and the details of which can be found in ref. ^40^. ORF-RATER and riboHMM only provide one strategy for ORF prediction. ORF-RATER is based on the linear regression method and riboHMM uses a hidden Markov model. RiboHMM only predicts one ORF for each transcript. For the comparison of performance difference in candidate-based prediction between Ribo-TISH and RibORF, the TISs and ORFs in the positive and negative sets were used as the candidates to perform ROC analysis. For a fair comparison, the requirement of RPFs compatible with transcript structure was turned off in Ribo-TISH. The comparison of the computational efficiency of different methods were performed using 4 processors on one node from the Nautilus High-Performance Computing cluster at UT MD Anderson Cancer Center, which is equipped with Intel Xeon E5-2680 processors running at 2.5 GHz and is running the Red Hat Linux 4.4.7.

### Functional enrichment analysis

The GO enrichment analysis was performed in DAVID (http://david.ncifcrf.gov). The genes with significantly increased TI efficiency under amino-acid deprivation condition were taken as input gene set, and the genes with RPKM ≥ 1 were used as the background.

### ORF prediction by integrating TI-seq and rRibo-seq data

Both AUG and near-cognate start codons were allowed in the prediction of novel ORFs. The *p*-value of the one-tailed NB test (*T*_*p*_) on the TI-seq data and the *p*-value of the frame test (*r*_*p*_) on the rRibo-seq data were combined using Fisher’s method. Multiple testing corrections were performed using the Benjamini–Hochberg Procedure^80^. The candidate novel ORFs were selected by the following criterions: *q*-value ≤ 0.05, *T*_*p*_ ≤ 0.01, and *r*_*p*_ ≤ 0.01. The predicted ORFs that had any overlap with the annotated ones in the same translation frame or were from pseudogenes were filtered out in downstream analysis. To predict the ORFs encoded by the annotated introns of PCGs, we extract consensus intron regions from Ensembl gene annotation. The candidate lncRNA-encoded smORFs that were selected for experimental validation were required to meet the following two criterions. First, the selected smORF-encoding human lncRNAs should have a homolog in at least one non-human primate species, based on the published assignment of lncRNA homologs^61–63^. Second, the smORFs from the homologous lncRNAs should share statistically significant similarity in amino-acid sequence (*E*-value <1 × 10^−10^), based on the BLAST analysis.

### Software and code availability

The webpage of Ribo-TISH toolkit can be found at the website of Department of Bioinformatics and Computational Biology at UT MD Anderson Cancer Center (http://bioinformatics.mdanderson.org/main/Ribo-TISH). Ribo-TISH package was written in Python and can be downloaded from Github (http://github.com/zhpn1024/ribotish).

### Data availability

The ribo-seq data were deposited to GEO (GSE94460).

## Electronic supplementary material

Supplementary Information

## References

[1] Sonenberg N, Hinnebusch AG (2009). Regulation of translation initiation in eukaryotes: mechanisms and biological targets. Cell.

[2] Curtis D, Lehmann R, Zamore PD (1995). Translational regulation in development. Cell.

[3] Buffington SA, Huang W, Costa-Mattioli M (2014). Translational control in synaptic plasticity and cognitive dysfunction. Annu. Rev. Neurosci..

[4] Spriggs KA, Bushell M, Willis AE (2010). Translational regulation of gene expression during conditions of cell stress. Mol. Cell.

[5] Starck SR (2016). Translation from the 5′ untranslated region shapes the integrated stress response. Science.

[6] Flygare J, Karlsson S (2007). Diamond-Blackfan anemia: erythropoiesis lost in translation. Blood.

[7] Scheper GC, van der Knaap MS, Proud CG (2007). Translation matters: protein synthesis defects in inherited disease. Nat. Rev. Genet..

[8] Silvera D, Formenti SC, Schneider RJ (2010). Translational control in cancer. Nat. Rev. Cancer.

[9] Ingolia NT (2014). Ribosome profiling reveals pervasive translation outside of annotated protein-coding genes. Cell Rep..

[10] Bazzini AA (2014). Identification of small ORFs in vertebrates using ribosome footprinting and evolutionary conservation. EMBO J..

[11] Aspden JL (2014). Extensive translation of small open reading frames revealed by poly-ribo-seq. eLife.

[12] Ji Z, Song R, Regev A, Struhl K (2015). Many lncRNAs, 5′UTRs, and pseudogenes are translated and some are likely to express functional proteins. eLife.

[13] Chew GL (2013). Ribosome profiling reveals resemblance between long non-coding RNAs and 5′ leaders of coding RNAs. Development.

[14] Rinn JL, Chang HY (2012). Genome regulation by long noncoding RNAs. Annu. Rev. Biochem..

[15] Slavoff SA (2013). Peptidomic discovery of short open reading frame-encoded peptides in human cells. Nat. Chem. Biol..

[16] Magny EG (2013). Conserved regulation of cardiac calcium uptake by peptides encoded in small open reading frames. Science.

[17] Nelson BR (2016). A peptide encoded by a transcript annotated as long noncoding RNA enhances SERCA activity in muscle. Science.

[18] Pauli A (2014). Toddler: an embryonic signal that promotes cell movement via Apelin receptors. Science.

[19] Anderson DM (2015). A micropeptide encoded by a putative long noncoding RNA regulates muscle performance. Cell.

[20] Jackson RJ, Hellen CU, Pestova TV (2010). The mechanism of eukaryotic translation initiation and principles of its regulation. Nat. Rev. Mol. Cell Biol..

[21] Lee S (2012). Global mapping of translation initiation sites in mammalian cells at single-nucleotide resolution. Proc. Natl Acad. Sci. USA.

[22] Ingolia NT, Lareau LF, Weissman JS (2011). Ribosome profiling of mouse embryonic stem cells reveals the complexity and dynamics of mammalian proteomes. Cell.

[23] Gao X (2015). Quantitative profiling of initiating ribosomes in vivo. Nat. Methods.

[24] Brar GA, Weissman JS (2015). Ribosome profiling reveals the what, when, where and how of protein synthesis. Nat. Rev. Mol. Cell Biol..

[25] Ingolia NT (2016). Ribosome footprint profiling of translation throughout the genome. Cell.

[26] Kochetov AV, Sarai A, Rogozin IB, Shumny VK, Kolchanov NA (2005). The role of alternative translation start sites in the generation of human protein diversity. Mol. Genet. Genomics.

[27] Oyama M (2007). Diversity of translation start sites may define increased complexity of the human short ORFeome. Mol. Cell. Proteomics.

[28] Fritsch C (2012). Genome-wide search for novel human uORFs and N-terminal protein extensions using ribosomal footprinting. Genome Res..

[29] Michel AM (2012). Observation of dually decoded regions of the human genome using ribosome profiling data. Genome Res..

[30] Xu H (2010). Length of the ORF, position of the first AUG and the Kozak motif are important factors in potential dual-coding transcripts. Cell Res..

[31] Van Damme P, Gawron D, Van Criekinge W, Menschaert G (2014). N-terminal proteomics and ribosome profiling provide a comprehensive view of the alternative translation initiation landscape in mice and men. Mol. Cell. Proteomics.

[32] Peabody DS (1989). Translation Initiation at non-Aug triplets in mammalian-cells. J. Biol. Chem..

[33] Wan J, Qian SB (2014). TISdb: a database for alternative translation initiation in mammalian cells. Nucleic Acids Res..

[34] Legendre R, Baudin-Baillieu A, Hatin I, Namy O (2015). RiboTools: a Galaxy toolbox for qualitative ribosome profiling analysis. Bioinformatics.

[35] Olshen AB (2013). Assessing gene-level translational control from ribosome profiling. Bioinformatics.

[36] Zhong Y (2017). RiboDiff: detecting changes of mRNA translation efficiency from ribosome footprints. Bioinformatics.

[37] Larsson O, Sonenberg N, Nadon R (2011). anota: Analysis of differential translation in genome-wide studies. Bioinformatics.

[38] Larsson O, Sonenberg N, Nadon R (2010). Identification of differential translation in genome wide studies. Proc. Natl Acad. Sci. USA.

[39] Xiao Z, Zou Q, Liu Y, Yang X (2016). Genome-wide assessment of differential translations with ribosome profiling data. Nat. Commun..

[40] Calviello L (2016). Detecting actively translated open reading frames in ribosome profiling data. Nat. Methods.

[41] Fields AP (2015). A regression-based analysis of ribosome-profiling data reveals a conserved complexity to mammalian translation. Mol. Cell.

[42] Raj, A. et al. Thousands of novel translated open reading frames in humans inferred by ribosome footprint profiling. *eLife* **5**, e13328 (2016). 10.7554/eLife.13328 PMC4940163 27232982

[43] Crappe J (2015). PROTEOFORMER: deep proteome coverage through ribosome profiling and MS integration. Nucleic Acids Res..

[44] Chung BY (2015). The use of duplex-specific nuclease in ribosome profiling and a user-friendly software package for Ribo-seq data analysis. RNA.

[45] Stumpf CR, Moreno MV, Olshen AB, Taylor BS, Ruggero D (2013). The translational landscape of the mammalian cell cycle. Mol. Cell..

[46] Andreev DE (2017). Insights into the mechanisms of eukaryotic translation gained with ribosome profiling. Nucleic Acids Res..

[47] Jackson R, Standart N (2015). The awesome power of ribosome profiling. RNA.

[48] Gerashchenko MV, Gladyshev VN (2014). Translation inhibitors cause abnormalities in ribosome profiling experiments. Nucleic Acids Res..

[49] O’Connor PB, Andreev DE, Baranov PV (2016). Comparative survey of the relative impact of mRNA features on local ribosome profiling read density. Nat. Commun..

[50] Rubio CA (2014). Transcriptome-wide characterization of the eIF4A signature highlights plasticity in translation regulation. Genome Biol..

[51] Rashid NU, Giresi PG, Ibrahim JG, Sun W, Lieb JD (2011). ZINBA integrates local covariates with DNA-seq data to identify broad and narrow regions of enrichment, even within amplified genomic regions. Genome Biol..

[52] Uren PJ (2012). Site identification in high-throughput RNA-protein interaction data. Bioinformatics.

[53] Akaike H (1974). A new look at the statistical model identification. IEEE Trans. Autom. Control.

[54] Schwarz G (1978). Estimating the dimension of a model. Ann. Stat..

[55] Michel AM, Andreev DE, Baranov PV (2014). Computational approach for calculating the probability of eukaryotic translation initiation from ribo-seq data that takes into account leaky scanning. BMC Bioinformatics.

[56] Robinson MD, Oshlack A (2010). A scaling normalization method for differential expression analysis of RNA-seq data. Genome Biol..

[57] Ashburner M (2000). Gene ontology: tool for the unification of biology. The Gene Ontology Consortium. Nat. Genet..

[58] Johnson MA (2014). Amino acid starvation has opposite effects on mitochondrial and cytosolic protein synthesis. PLoS ONE.

[59] Wethmar K, Barbosa-Silva A, Andrade-Navarro MA, Leutz A (2014). uORFdb–a comprehensive literature database on eukaryotic uORF biology. Nucleic Acids Res..

[60] Schueler M (2014). Differential protein occupancy profiling of the mRNA transcriptome. Genome Biol..

[61] Necsulea A (2014). The evolution of lncRNA repertoires and expression patterns in tetrapods. Nature.

[62] Washietl S, Kellis M, Garber M (2014). Evolutionary dynamics and tissue specificity of human long noncoding RNAs in six mammals. Genome Res..

[63] Hezroni H (2015). Principles of long noncoding RNA evolution derived from direct comparison of transcriptomes in 17 species. Cell Rep..

[64] Pauli A, Valen E, Schier AF (2015). Identifying (non-)coding RNAs and small peptides: challenges and opportunities. BioEssays.

[65] Tani H, Torimura M, Akimitsu N (2013). The RNA degradation pathway regulates the function of GAS5 a non-coding RNA in mammalian cells. PLoS ONE.

[66] Bicknell AA, Cenik C, Chua HN, Roth FP, Moore MJ (2012). Introns in UTRs: why we should stop ignoring them. BioEssays.

[67] Cenik C (2011). Genome analysis reveals interplay between 5′UTR introns and nuclear mRNA export for secretory and mitochondrial genes. PLoS Genet..

[68] Robinson MD, McCarthy DJ, Smyth GK (2010). edgeR: a Bioconductor package for differential expression analysis of digital gene expression data. Bioinformatics.

[69] Love MI, Huber W, Anders S (2014). Moderated estimation of fold change and dispersion for RNA-seq data with DESeq2. Genome Biol..

[70] Wang X, Hou J, Quedenau C, Chen W (2016). Pervasive isoform-specific translational regulation via alternative transcription start sites in mammals. Mol. Syst. Biol..

[71] Floor, S. N. & Doudna, J. A. Tunable protein synthesis by transcript isoforms in human cells. *eLife* **5**, e10921 (2016). 10.7554/eLife.10921 PMC4764583 26735365

[72] Guttman M (2010). Ab initio reconstruction of cell type-specific transcriptomes in mouse reveals the conserved multi-exonic structure of lincRNAs. Nat. Biotechnol..

[73] Jiang H, Wong WH (2009). Statistical inferences for isoform expression in RNA-Seq. Bioinformatics..

[74] Li W, Jiang T (2012). Transcriptome assembly and isoform expression level estimation from biased RNA-Seq reads. Bioinformatics..

[75] Trapnell C (2010). Transcript assembly and quantification by RNA-Seq reveals unannotated transcripts and isoform switching during cell differentiation. Nat. Biotechnol..

[76] Nicolae M, Mangul S, Mandoiu II, Zelikovsky A (2011). Estimation of alternative splicing isoform frequencies from RNA-Seq data. Algorithms Mol. Biol..

[77] Li B, Dewey CN (2011). RSEM: accurate transcript quantification from RNA-Seq data with or without a reference genome. BMC Bioinformatics.

[78] Cenik C, Derti A, Mellor JC, Berriz GF, Roth FP (2010). Genome-wide functional analysis of human 5′ untranslated region introns. Genome Biol..

[79] Dobin A (2013). STAR: ultrafast universal RNA-seq aligner. Bioinformatics.

[80] Benjamini Y, Hochberg Y (1995). Controlling the false discovery rate—a practical and powerful approach to multiple testing. J. Roy. Stat. Soc. B Met..

