# Supplementary material for: Genome-wide identification and differential analysis of translational initiation
Source: Nat Commun. 2017 Nov 23;8:1749. doi: 10.1038/s41467-017-01981-8 (PMC5701008; doi:10.1038/s41467-017-01981-8)
Supplement: Supplementary file 1 — Supplementary Information [file 41467_2017_1981_MOESM1_ESM.pdf]

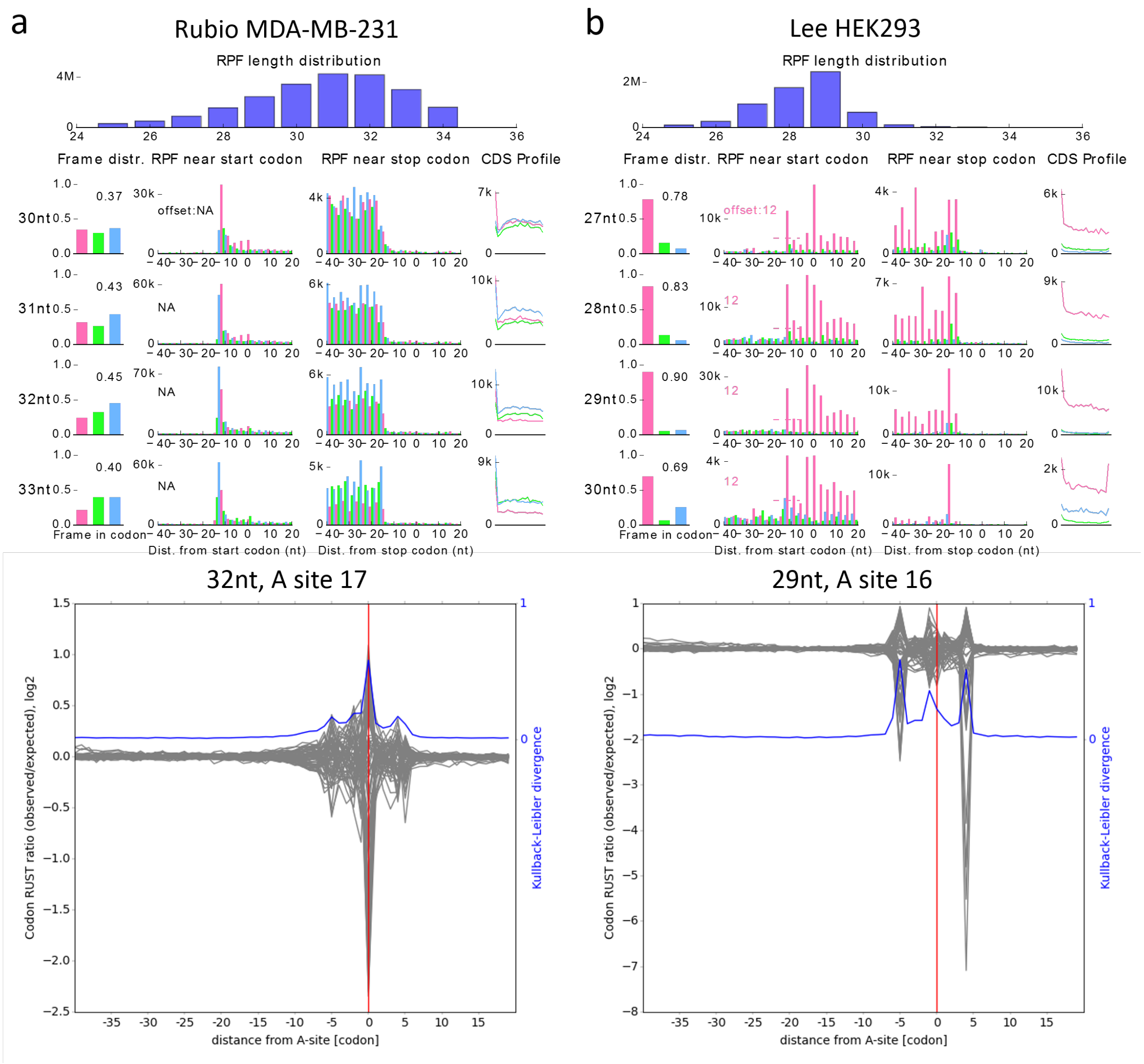

Supplementary Figure 1. The difference in the quality control used by Ribo-TISH and RUST. Quality control (QC) with Ribo-TISH (upper panel) and RUST (lower panel) for (a) an rRibo-seq dataset (SRR1573934) shown in the RUST paper and (b) an rRibo-seq data used in our analysis. In the QC figures generated by RUST, the grey curves represent individual RUST ratio values of 61 codons across the mRNA. The corresponding Kullback–Leibler divergence (K–L) is shown in blue. High K-L divergence at positions other than A-site (red line) may represent biases or artifacts introduced by different experimental protocols.

Group	Criterion	Poisson	ZIP	NB	ZINB	Best
0	AIC	1542404	1482546	1478173	1478178	NB
	BIC	1542418	1482573	1478200	1478219	NB
1	AIC	987755.2	943860.2	934057.5	934071.4	NB
	BIC	987767.1	943884.1	934081.3	934107.2	NB
2	AIC	795937.1	743560.3	725710.4	725732.7	NB
	BIC	795948.4	743582.9	725732.9	725766.5	NB
3	AIC	659714.6	599324.9	572039.6	572070.5	NB
	BIC	659725.4	599346.3	572061	572102.6	NB
4	AIC	528720.1	460934	416663.8	416701.9	NB
	BIC	528730.2	460954.2	416684.1	416732.2	NB
5	AIC	439251.6	366319.6	302291.4	302331.7	NB
	BIC	439261.1	366338.7	302310.4	302360.3	NB
6	AIC	317587.9	249925.1	174295.4	174321.9	NB
	BIC	317596.6	249942.6	174312.9	174348.1	NB
7	AIC	260530.4	199467.9	104575.1	104593	NB
	BIC	260538.5	199483.9	104591.1	104617	NB
8	AIC	221384.8	176606.8	56771.3	56783.28	NB
	BIC	221392	176621.1	56785.57	56804.69	NB
9	AIC	87877.91	67263.68	20064.88	20068.16	NB
	BIC	87883.93	67275.72	20076.93	20086.23	NB

Supplementary Table 1. The AIC and BIC values for TIS background model selection (ZIP: Zero Inflated Poisson, NB: Negative Binomial, ZINB: Zero Inflated Negative Binomial) using a published LTM-based TI-seq dataset. The AIC and BIC values are shown for different groups of transcripts, which are stratified based on their TI-seq signal density (group 0 corresponding to the lowest ones and group 9 corresponding to the highest ones).

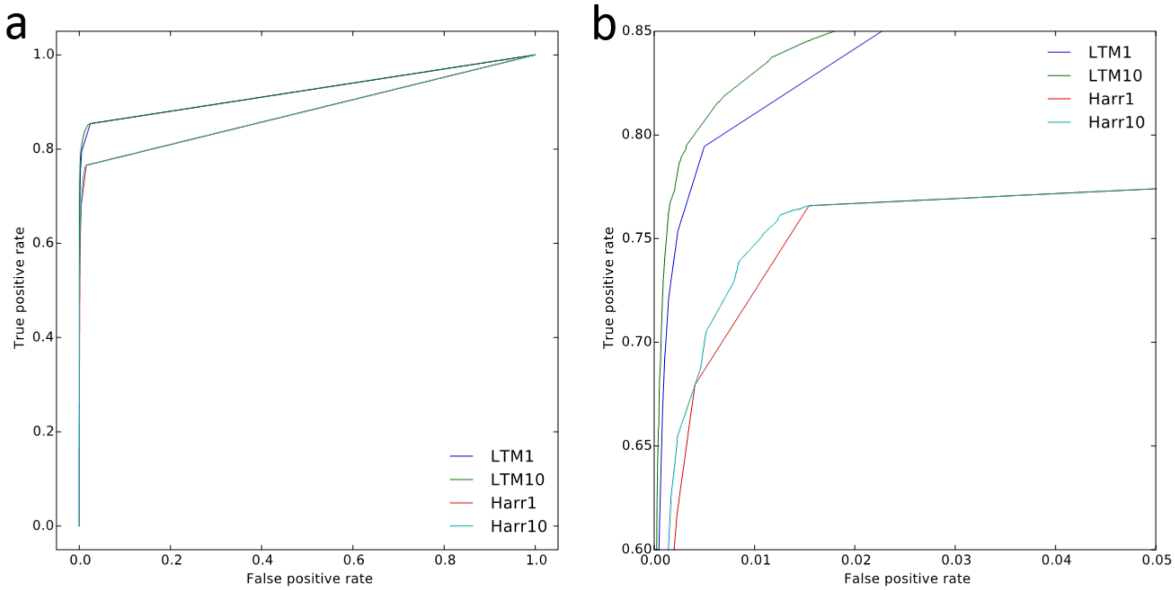

Supplementary Figure 2. The performance of TIS prediction from LTM- or Harr-based TI-seq data. A comparison of TIS prediction performance between Harr-based and LTM-based TI-seq data using a single background distribution (Harr1 and LTM1) for all transcripts or group-specific background distributions for ten groups with different TI-seq signal density (Harr10 and LTM10). The full-scale (a) and zoomed (b) ROC curves are shown. An RPKM value of 10 was used as the cutoff to define the actively translating genes for the positive TIS sets.

Data type & Number of groups	AUC	pAUC (FPR=0.05)
LTM 1 group	0.91693	0.75971
LTM 10 groups	0.91841	0.78577
Harr 1 group	0.87570	0.70007
Harr 10 groups	0.87637	0.71329

Supplementary Table 2. The total AUC and partial AUC (pAUC) at the false positive rate (FPR) of 5% are shown for TIS prediction using LTM-based or Harr-based TI-Seq data. A single background distribution (Harr1 and LTM1) or group-specific background distributions for ten groups with different TI-seq signal density (Harr10 and LTM10) was used.

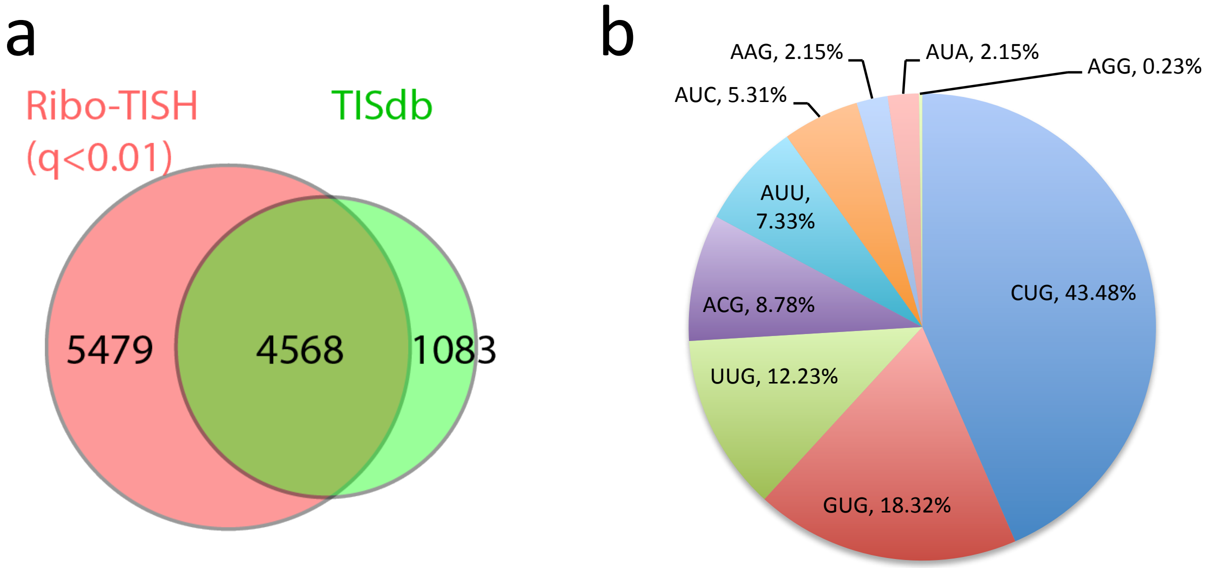

Supplementary Figure 3. The TISs predicted by Ribo-TISH. (a) The overlap of predicted AUG or near-cognate TISs by Ribo-TISH from an LTM-based TI-seq dataset (SRR618772 and SRR618773) in HEK293 cells with those collected in TISdb. (b) The frequency (%) of different near-cognate codon usage at all TISs predicted by Ribo-TISH.

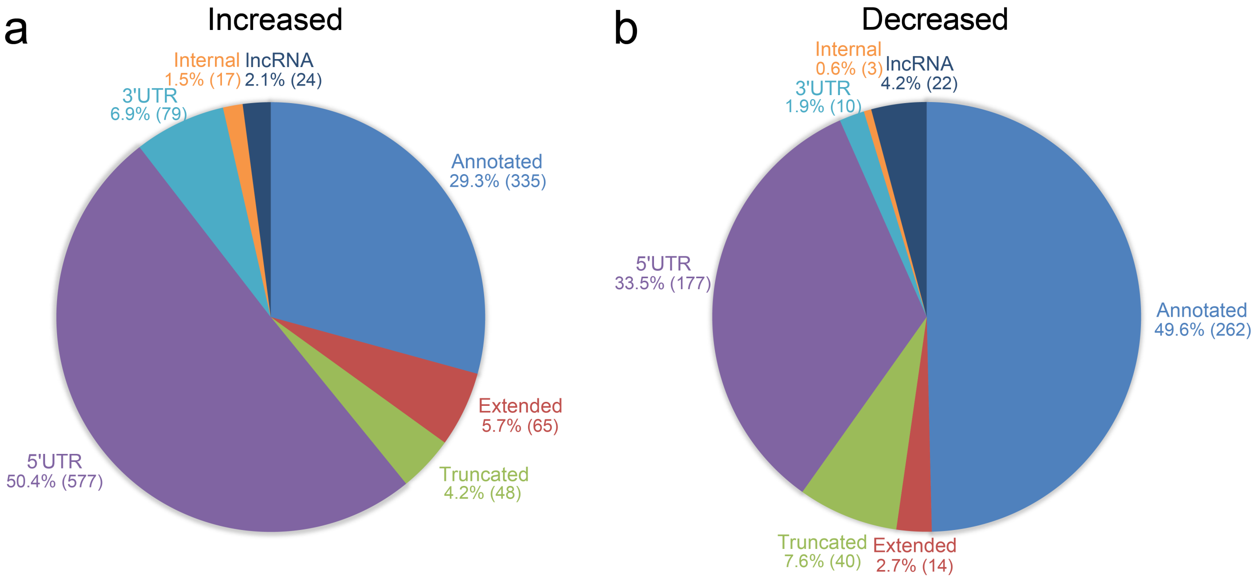

Supplementary Figure 4. The distribution of differential TISs over different categories. Pie charts show the distribution of the non-redundant TISs that were identified by Ribo-TISH and exhibited (a) increased and (b) decreased TI efficiency upon amino acid starvation.

Functionalities	Ribo-TISH	RibORF	RiboTaper	ORF-RATER	riboHMM
Predict TIS from TI-Seq data	Y	N	N	N	N
Differential TIS identification	Y	N	N	N	N
Multiprocessing	Y	N	Y	Y	N
De novo ORF prediction	Y	N	Y	Y	Y
User specified candidates	Y	Y	N	N	N
Support Non-AUG start	Y	NA	N	Y	Y
P-site for spliced reads	Y	N	Y	Y	Y
Reads compatible with splice junctions	Y	N	N	N	N

Supplementary Table 3. The functionalities supported by different computational tools, including Ribo-TISH, RibORF, RiboTaper. ORF-RATER, and riboHMM.

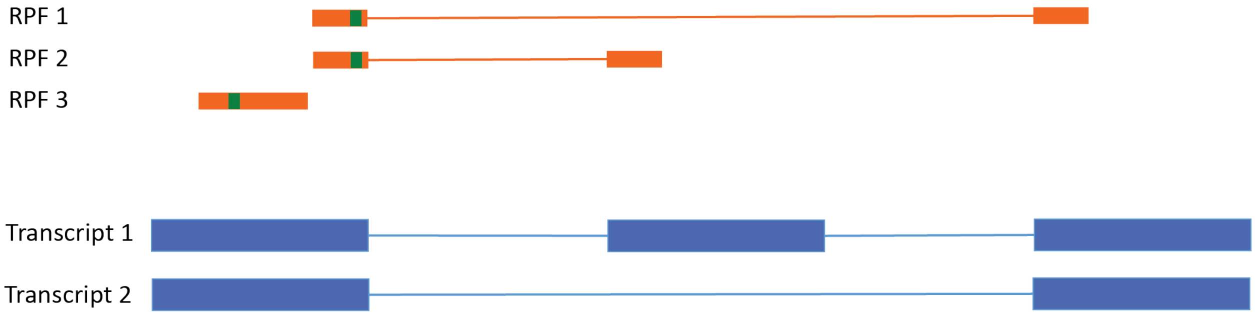

	Transcript 1	Transcript 2
Not require compatible	1,2,3	1,2,3
Compatible (only in Ribo-TISH)	2,3	1,3

Supplementary Figure 5. Assessing the compatibility of RPF reads with transcript structure. In this illustration, there are 3 RPF reads (orange) that are associated with 2 transcripts (blue), with the P-sites of RPFs being shown in green. The other tools assign a RPF to a transcript as long as its P-site (green) is inside the exon region of the transcript. In contrast, Ribo-TISH enforces the compatibility of the whole RPF reads with the transcript exon-intron structure. For example, Ribo-TISH does not assign RPF 1 to transcript 1 because the RPF 1 covers the 1st and 3rd exon, but not the 2nd exon of transcript 1. Similarly, the RPF 2 is only compatible with the exon-intron structure of transcript 1, but not transcript 2. RPF 3 can be assigned to both transcripts. The resultant assignment of the 3 RPF reads to the 2 transcripts with or without enforcing their compatibility with transcript structure is shown in the table in the lower panel.

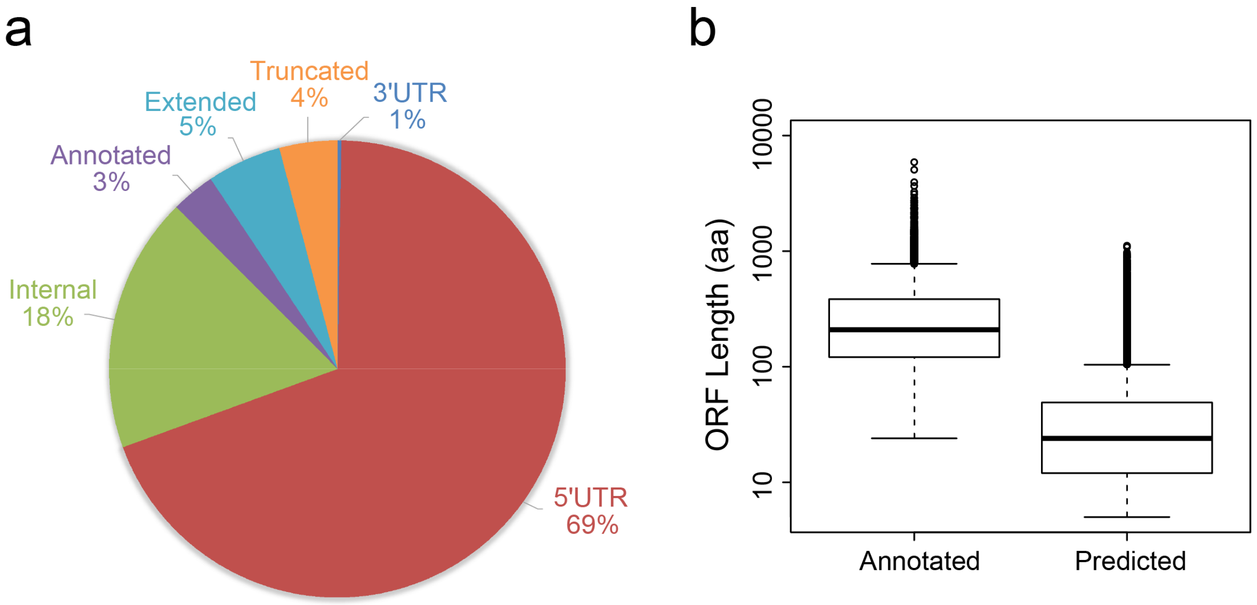

Supplementary Figure 6. The features of the riboHMM predicted ORFs. (a) The distribution across different types of ORFs and (b) the boxplot-based length distribution of the ORFs that were predicted by riboHMM.

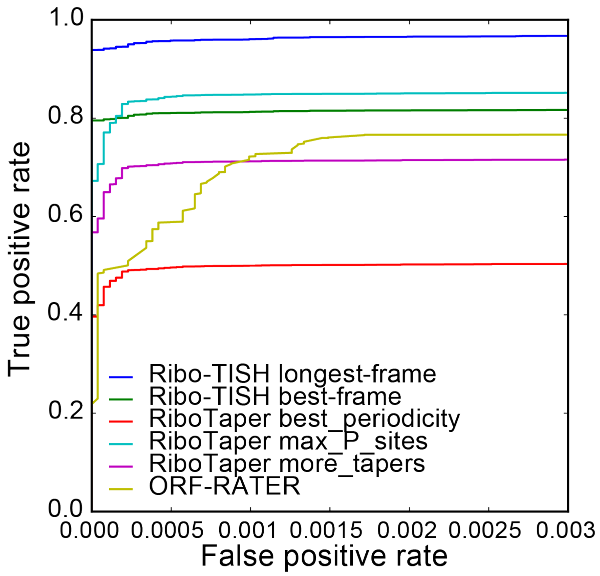

Supplementary Figure 7. Comparing the performance of different methods in ORF prediction. A comparison of ROC curves across six strategies of ORF prediction that were implemented in Ribo-TISH, RiboTaper and ORF-RATER. RPKM value of 10 was used as the cutoff to define the actively translating genes.

	Method	AUC	pAUC (FPR=0.01)	pAUC (FPR=0.05)
RPKM>=10	Ribo-TISH longest-frame	0.98416	0.96579	0.96861
	Ribo-TISH best-frame	0.90827	0.81630	0.82092
	RiboTaper best_periodicity	0.75101	0.50276	0.51414
	RiboTaper max_P_sites	0.92576	0.84933	0.85469
	RiboTaper more_tapers	0.85755	0.71412	0.72178
	ORF-RATER	0.88269	0.74743	0.76773
RPKM >= 1	Ribo-TISH longest-frame	0.98396	0.96464	0.96838
	Ribo-TISH best-frame	0.87130	0.74238	0.74876
	RiboTaper best_periodicity	0.72532	0.45140	0.46400
	RiboTaper max_P_sites	0.85283	0.70439	0.71252
	RiboTaper more_tapers	0.78176	0.56338	0.57401
	ORF-RATER	0.83772	0.64310	0.67731

Supplementary Table 4. The total AUC and partial AUC (pAUC) at 1% and 5% false positive rate (FPR) are shown for Ribo-TISH, RiboTaper and ORF-RATER to compare their performance in ORF prediction from rRiboseq data, using different RPKM cutoffs to define active translation.

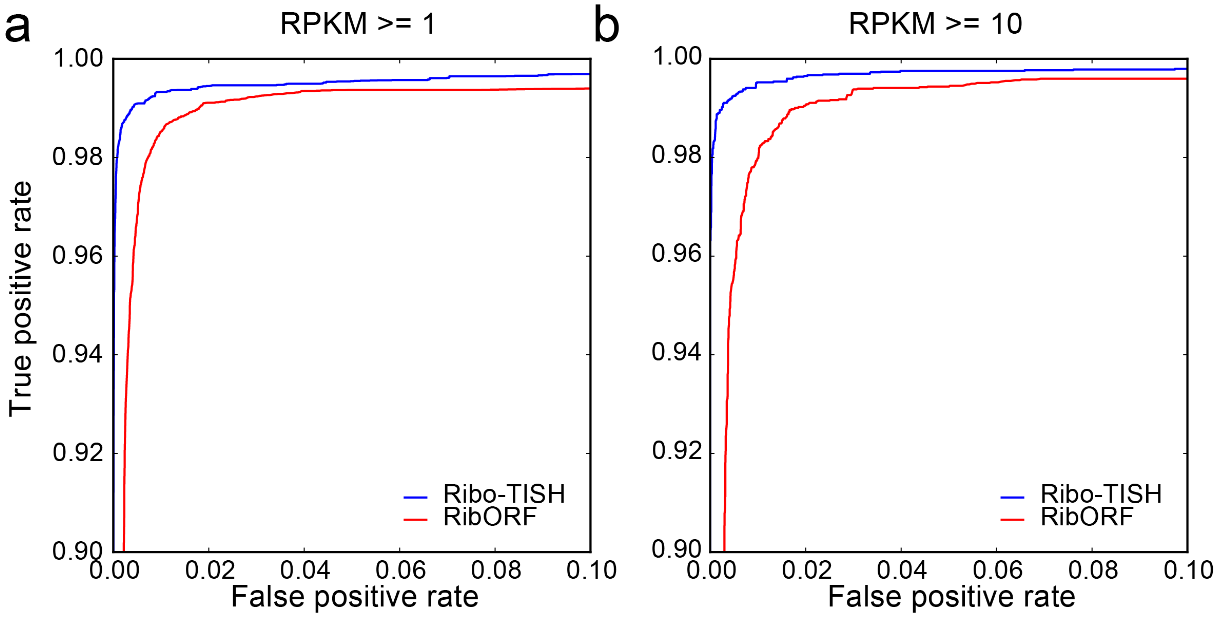

Supplementary Figure 8. Comparing the performance of two methods in candidate-based ORF prediction. Comparison of ROC curves of candidate-based ORF prediction that were implemented in Ribo-TISH and RibORF. RPKM values of (a) 1 and (b) 10 were used as a cutoff to define the actively translating genes.

	Method	AUC	pAUC (FPR=0.01)	pAUC (FPR=0.05)
RPKM >= 10	Ribo-TISH	0.99861	0.99083	0.99563
	RibORF	0.99566	0.80545	0.95445
RPKM >= 1	Ribo-TISH	0.99849	0.98771	0.99323
	RibORF	0.99561	0.86830	0.96701

Supplementary Table 5. The total AUC and partial AUC (pAUC) at 1% and 5% FPR are shown for Ribo-TISH and RibORF to compare their performance in user-specified-candidate-based prediction of ORFs from rRibo-seq data, using different RPKM cutoffs to define active translation.

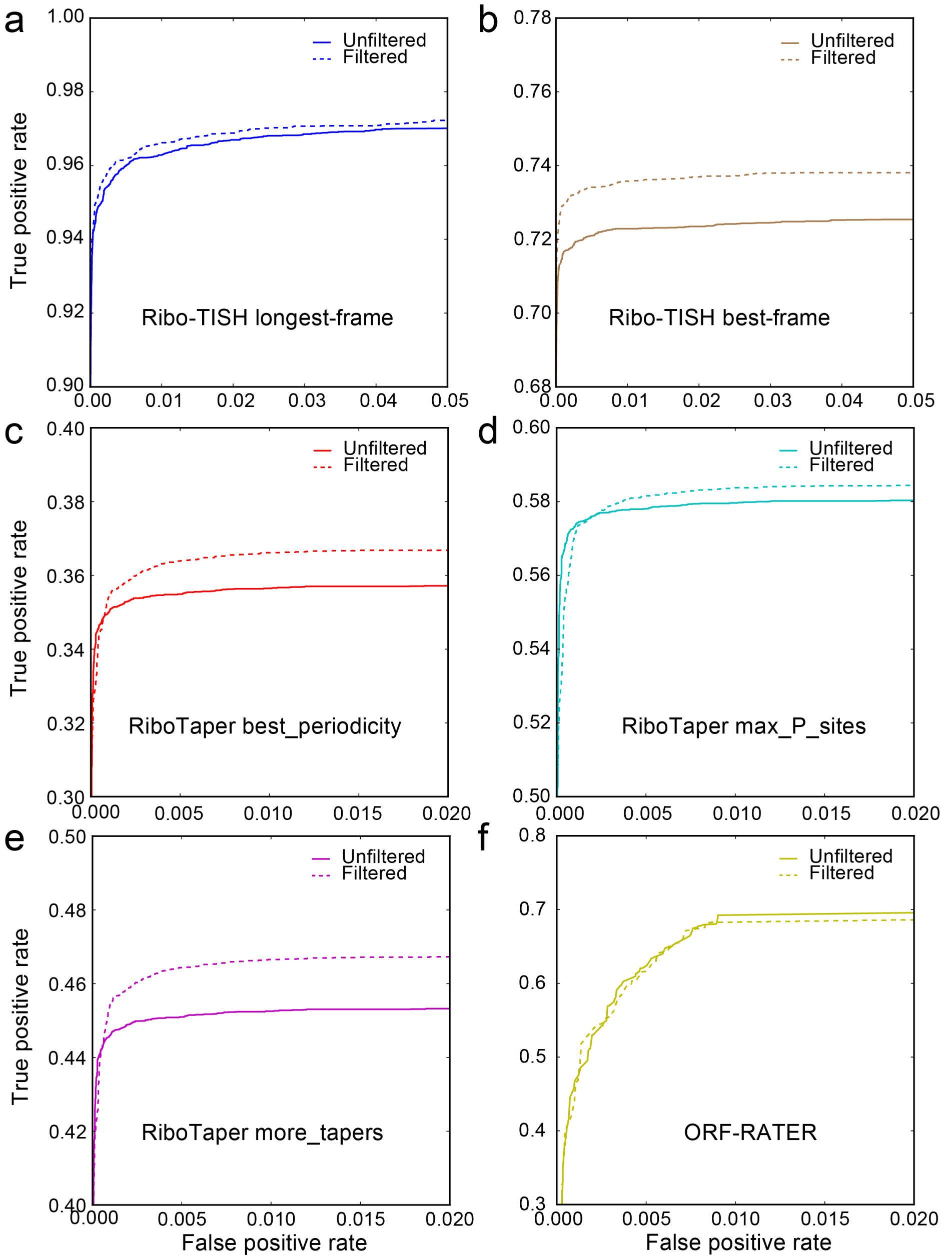

Supplementary Figure 9. The effect of filtering low quality RPF reads on ORF prediction. A comparison of ROC curves before and after filtering of low quality reads (31 nt reads in **Fig. 2c**) across six strategies of ORF prediction from rRibo-seq data, including (a) the Ribo-TISH longest-frame, (b) Ribo-TISH best-frame, (c) RiboTaper best periodicity, (d) RiboTaper max_P_sites, (e) RiboTaper more tapers and (f) ORF-RATER. The positive set and negative set are the same as those in Figure 5b. An RPKM value of 1 was used as the cutoff to define the actively translating genes.

Tool	CPU time (s)	Run time (s)	Memory (MB)	Disk space for intermediate files (bytes)
Ribo-TISH	3,427	1,157	1,658	<1k
RiboTaper	169,722	40,005	68,682	15G
ORF-RATER	22,984	8,816	36,809	424M

Supplementary Table 6. A comparison of computational efficiency of Ribo-TISH, RiboTaper and ORF-RATER, in prediction of ORFs with AUG starts codons from rRibo-seq data. The comparison of CPU time, run time, memory and disk space for intermediate files was performed using a rRibo-seq dataset of 25M mapped reads and an RNA-seq dataset of 34M mapped reads (required only by RiboTaper) on 93,892 annotated protein coding transcripts in Ensembl version 83. The computation was carried in one node from the MD Anderson Nautilus High Performance Computing cluster, using 4 processors. Each node is equipped with Intel Xeon E5-2680 processors running at 2.5GHz and is running the Red Hat Linux 4.4.7.

Tool	CPU time (s)	Run time (s)	Memory (MB)	Disk space for intermediate files (bytes)
Ribo-TISH	11,980	4,106	5,644	<1k
ORF-RATER	>64,923	>16,782	>184,858	1.5G

Supplementary Table 7. A comparison of computational efficiency of Ribo-TISH and ORF-RATER (RiboTaper does not support NUG and other near-cognate start codons), in prediction of ORFs with NUG (N=A, U, C and G) start codons. The comparison of CPU time, run time, memory and disk space for intermediate files was performed using a rRibo-seq dataset of 25M mapped reads and 93,892 annotated protein coding transcripts in Ensembl version 83. The computation was carried in one node from the MD Anderson Nautilus High Performance Computing cluster using 4 processors. Each node is equipped with Intel Xeon E5-2680 processors running at 2.5GHz and is running the Red Hat Linux 4.4.7. ORF-RATER required more than 180 GB of CPU memory and encountered an issue of memory overflow.

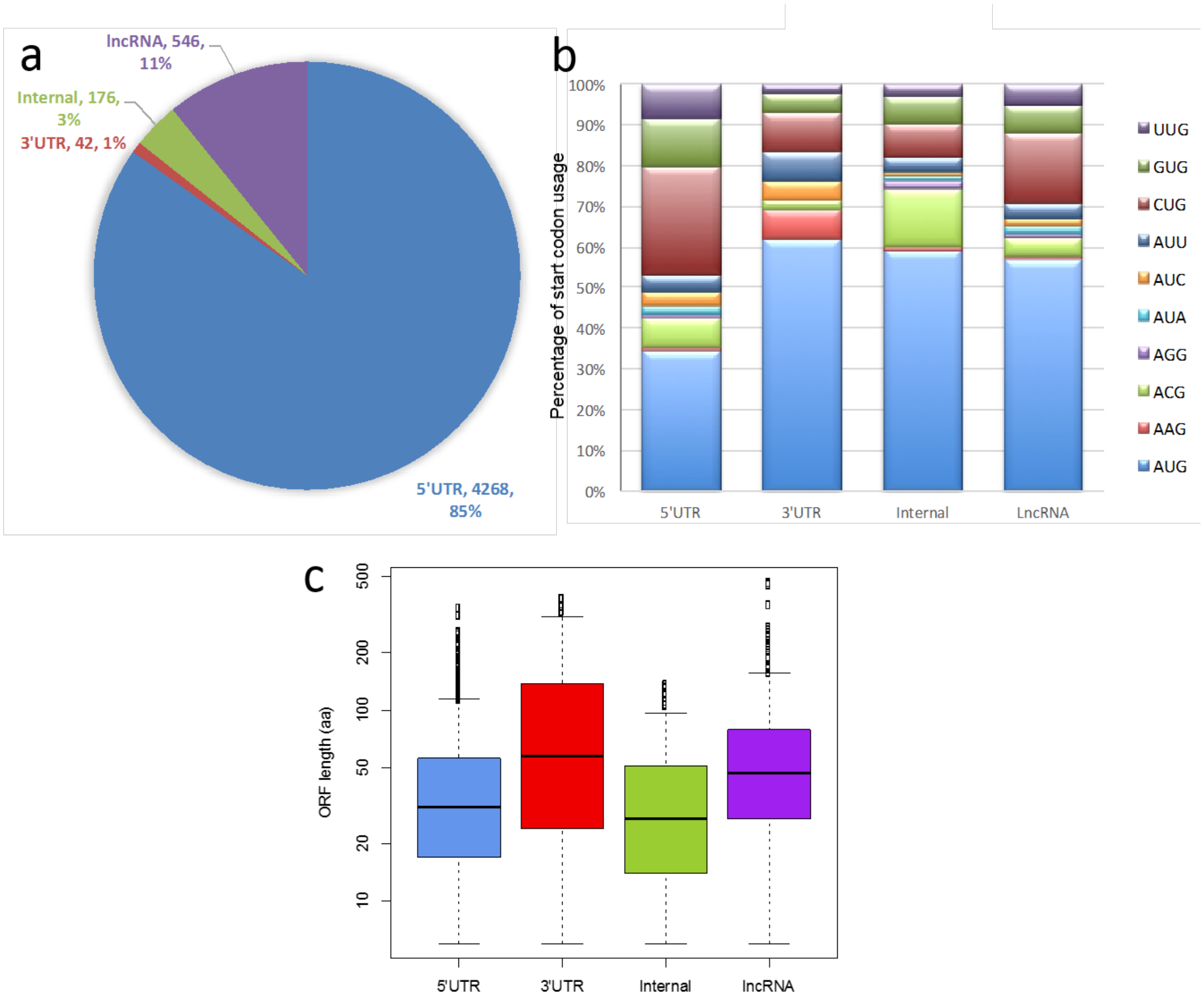

Supplementary Figure 10. The characteristics of novel ORFs predicted using TI-seq and rRibo-seq data. (a) The number of predicted new ORFs in different categories, including 5'UTR, 3'UTR, lncRNA and ORFs that are internal to and out-of-frame with the known ORFs (internal). (b) The percentage of start codon usage for ORFs in different categories. (c) The length distribution for ORFs in different categories.

	All	50-100aa	AUG	Conserved	Final
5'UTR	4268	1040	248	NA	248
lncRNA	546	180	99	14	14
intron	24	9	3	NA	3

Supplementary Table 8. Number of ORF candidates after each step of filtering.

Type	ID	Symbol	Genomic Locations	Len
5’UTR	ENST00000216554	*EIF5*	14:103334575-103335924:+	97
	ENST00000618250	*RHNO1*	12:2885284-2885512:+	75
	ENST00000574590	*KANSL1*	17:46171959-46223697:-	99
	ENST00000389063	*DCP2*	5:112976904-112985968:+	71
	ENST00000487519	*TFAM*	10:58385432-58386303:+	99
	ENST00000403825	*IFRD1*	7:112450486-112450645:+	52
	ENST00000357590	*SLC37A4*	11:119029332-119029530:-	65
	ENST00000443868	*MRS2*	6:24403000-24405171:+	79
	ENST00000314134	*SLC35C1*	11:45805361-45805604:+	80
	ENST00000524490	*C11orf30*	11:76445105-76453357:+	91
lncRNA	ENST00000442067	*GAS5*	1:173866180-173867041:-	50
	ENST00000411630	*DANCR*	4:52713673-52713853:+	59
	ENST00000591372	*LINC00665*	19:36331484-36331643:-	52
	ENST00000602414	*SNHG8*	4:118278819-118278978:+	52
	ENST00000501122	*NEAT1*	11:65422887-65423103:+	71
	ENST00000551421	*RP11-620J15.3*	12:57931609-57935902:-	63
	ENST00000580180	*LRRC75A-AS1*	17:16439054-16440252:+	53
	ENST00000597980	*CTD-2619J13.14*	19:58406714-58408343:-	90
	ENST00000545440	*SNHG1*	11:62853106-62855216:-	65
	ENST00000499346	*LINC01184*	5:128082767-128083022:-	84
Intron	ENSG00000189114	*BLOC1S3*	19:45178865-45179120:+	84
	ENSG00000110958	*PTGES3*	12:56686634-56686841:-	68
	ENSG00000010030	*ETV7*	6:36363205-36363475:-	89

Supplementary Table 9. Top candidate smORFs from 5’UTRs, lncRNAs and intronic regions. The candidates selected for experimental validation were shown in red.

DANCR F primer:<br>CTAGTCTAGAAGAGTCTTCCCGGGATTGG
DANCR R primer with 3xFLAG:<br>CGCGGATCCTCACTTGTCGTCGTCGTCCTTGTAGTCGATGTCGTGGTCCTTGTAGTCACCGTCGTGG<br>TCCTTGTAGTCGGAATGCAGCTGCAGAGTATT
EIF5 F primer:<br>CTAGTCTAGATCTGGGAAAGGCGGGGGGA
EIF5 R primer with 3xFLAG:<br>CGCGGATCCTCACTTGTCGTCGTCGTCCTTGTAGTCGATGTCGTGGTCCTTGTAGTCACCGTCGTGG<br>TCCTTGTAGTCGACGGGGCATCTTGTAGCGA
DANCR gBlocks:<br>CTAGTCTAGA**AGAGTCTTCCCGGGATTGGCTGCGGGCCTCGCGACCCTCCTGCTTCCCTCCCCGCC**<br>**CCGCGCCGCCTCTCTGGTTTGTGCGCCCGTCGCAGGTCGCAGGCCTCTTTGTCAGCTGGAGTTGC**<br>**GCGGGCTGACGCGCCACTATGTAGCGGGTTTCGGGCGGGCCACGCGTGCGGGACAGGAACCCAA**<br>**CCCCAGCCGACCTTGAGCTCCAGGAGTTCGTCTCTTACGTCTGCGGAAGTGCAGCTGCCTCAGTT**<br>**CTTAGCGCAGGTTGACAACTACAGGCACAAGCCATTGAAGCTGGAATGTCCTGTTGCTGGTATTTC**<br>**AATTGACTTAAGCCAACTATCCCTTCAGTTACAATAGGAAAGTGCCTCTAATAAGGCCAAATATGC**<br>**GTACTAACTTGTAGCAACCACGTGTCCGTGCAGTGCCACAGGAGCTAGAGCAGTGACAATGCTGG**<br>**TGGCAACAGGGCAGTGTAGCAGGTGCTTCATGTTCACCTTTTCAACCTTTTCATTTAATTGTCACAA**<br>**CTCGGAGGTGGATTCTGTTAGGGACAGGCTGCCCCAGGACCACTCCGCCCCCGCTAACTCAATGC**<br>**AGCTGACCCTTACCCTGAATACTCTGCAGCTGCATTCC**GACTACAAAGACGATGACGACAAGTGAG<br>GATCCGCG
EIF5 gBlocks:<br>CTAGTCTAGA**TCTGGGAAAGGCGGGGGGAGGGGAGAGGAAGGAAGCGAGACCGGGGCTGGAGG**<br>**CGGGCCGAGGCCGCGAGTTGAGCCTCAGGAAGCAGAAGGGGCGCGGAAAGATGCGATTGACGT**<br>**CGCAAGCGAACCAATGACGAGCGAGCTGTGGCAGGCCTCCAGCCAATGGGCAGTGAGGCCTGAC**<br>**GCGGGGGGCGGACGCTGGGGCCGAGGGTAGCTTGAGCGCGGCGGCGGCGTTGTTCAGTCAGAG**<br>**CGAGAACATTCCAGAGGTCGCCCAGCTCCGGCGCTGACGGGTGTGGACCGCGGACGTCGCTGGG**<br>**ACAGCCCCTCCCCGCTGCTCGGCGGCGGCACCTGGCCCGGCCGCTCCTCGCTGCGCTTCGCCTC**<br>**CGCCTCCTCGGACTCGGACTCGGGTTTATATCGCGCCTCACTTCATCCCAGTCCCGGGCGAGCAG**<br>**CGTTGGGTTTATGTCTTTATTTGACGAAAACGAGCTGTTGCGCAGCCATTGGTACCTGTATTGGGG**<br>**AAACATAGCATACAAGCAAGAAGCTTACAGCCTCAGTGGCGAAAATTTTTTCATGTCAGAGACCG**<br>**AGAACTCTTGCAGTCGTTTATGTCATCCCTTCTTCTCCAGACAGAAGATACCAAAAAGTTGCAATC**<br>**AAAGATCTCTTCATCTTATTGATAAAGCCACTAATAAGCCAAAATGTCTGTCAATGTCAACCGCAG**<br>**CGTGTCAGACCAGTTCTATCGCTACAAGATGCCCCGTC**GACTACAAAGACGATGACGACAAGTGAG<br>GATCCGCG
BLOC1S3 gBlocks:<br>CTAGTCTAGA**GAAATCACAGCCCTTCAGCTGCCACGGTGAGAACGCAGCACTCGGGTTAGGAAGC**<br>**GGATCTCGCAAGCTCCGAGCGTCAGCTGCCGGGTACGGTCTTTGGCGTTAGCGCTTCTCCCATCC**<br>**CATGAGTGCCCCCAGCAGAGTCCAGTCGGACTGTCATCCTTTCTGCGACTCTGGCGCTGGTCCCG**<br>**AGCAGCTCACGGGCCCGTGTGCTTCCGGGTCTGAGATTGGCATGGTGGACCAGGTGGAGGGTGT**<br>**GGCCTCTACTAGGAGGCAAATTCGTAAAGACCTCGGCTTGGGACTCCGGGAACTCGGGCCCCAG**<br>**ATCCTTGTTCGAGCTGGTCTTCAGTTTCCCCATCTGTACGCTGAAGAGCCTGGGGTCCAG**GACTAC<br>AAGGACCACGACGGTGACTACAAGGACCACGACATCGACTACAAGGACGACGACGACAAGTGAGGA<br>TCCGCG

Supplementary Table 10. The primer sequences for PCR amplification from synthesized gBlocks and the sequences of the synthesized gBlocks for smORF cloning. The 5' upsteam sequences and CDS of the smORFs were marked in bold. Underlined sequences are 3xFLAG or 1xFLAG tag sequences. The start codon and stop codon of the smORFs were marked in red.

1 MSAPSRVQSD CHPFCDSGAG PEQLTGPCAS GSEIGMVDQV EGVASTRRQI

51 R**KDLGLGLRE LGPQILVRAG LQFPHLYAEE PGVQDYKDHD GDYKDHDIDY**

101 **KDDDDK**

Query	Start	End	Observed	Mr(expt)	Mr(calc)	ppm	Miss	Score	Expect	Rank	Unique	Peptide
113	52	59	436.2721	870.5296	870.5287	1.08	1	44	4.20E-05	1	U	R.KDLGLGLR.E
114	52	59	436.2721	870.5296	870.5287	1.08	1	54	4.10E-06	1	U	R.KDLGLGLR.E
18	53	59	372.2237	742.4329	742.4337	-1.14	0	40	0.00011	1	U	K.DLGLGLR.E
19	53	59	372.2238	742.4331	742.4337	-0.87	0	46	2.70E-05	1	U	K.DLGLGLR.E
20	53	59	372.2246	742.4345	742.4337	1.1	0	47	2.10E-05	1	U	K.DLGLGLR.E
218	60	68	512.8106	1023.607	1023.608	-0.97	0	45	3.50E-05	1	U	R.ELGPQILVR.A
219	60	68	512.8153	1023.616	1023.608	8.21	0	45	3.30E-05	1	U	R.ELGPQILVR.A
662	69	87	721.3578	2161.052	2161.048	1.64	0	48	1.70E-05	1	U	R.AGLQFPHLYAEEPGVQDYK.D
697	88	106	775.3123	2322.915	2322.915	0.099	2	49	1.40E-05	1	U	K.DHDGDYKDHDIDYKDDDDK.-
551	95	106	747.3053	1492.596	1492.595	0.44	1	60	8.90E-07	1	U	K.DHDIDYKDDDDK.-
552	95	106	498.5401	1492.599	1492.595	2.06	1	45	3.40E-05	1	U	K.DHDIDYKDDDDK.-

Supplementary Table 11. *BLOC1S3* intronic smORF MS/MS protein sequence coverage**.** The peptides with matched spectrum were shown in bold red.

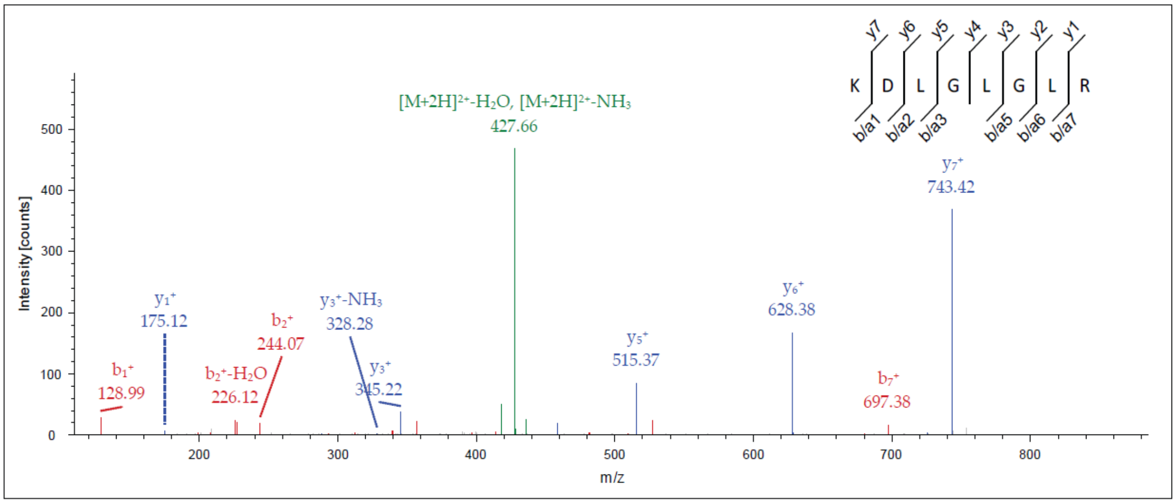

Supplementary Figure 11. The MS/MS spectrum that identifies the peptide KDLGLGLR in *BLOC1S3* intronic smORF. The IonScore for this peptide is 54 and the expectation Value is 8.1E-005. The monoisotopic (m/z) is 436.27209 Da (+0.44 mmu/+1.01 ppm).

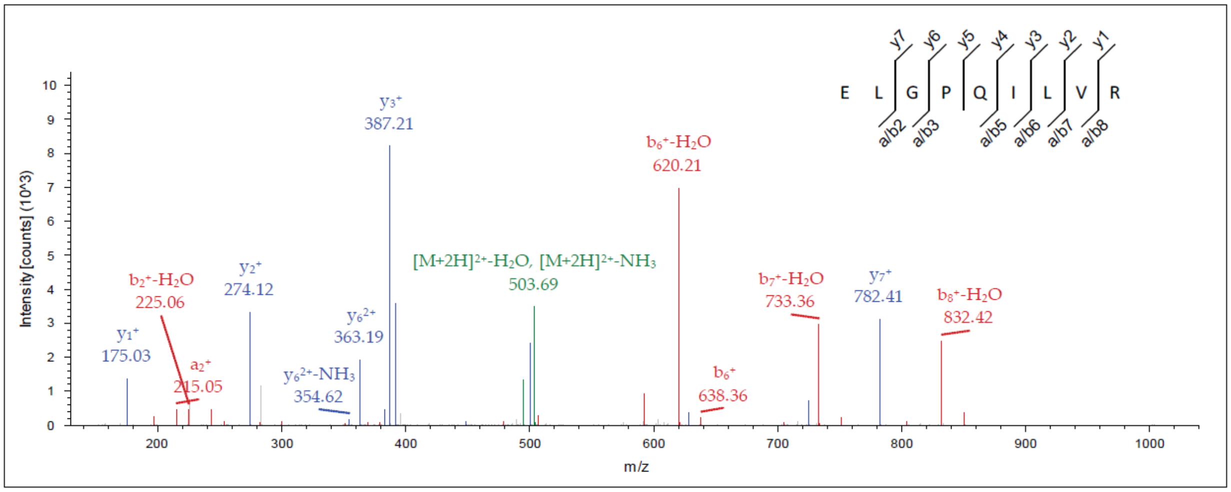

Supplementary Figure 12. The MS/MS spectrum that identifies the peptide ELGPQILVR in *BLOC1S3* intronic smORF. The IonScore for this peptide is 45 and the expectation Value is 6.6E-004. The monoisotopic (m/z) is 512.81531 Da (+4.17 mmu/+8.13 ppm).

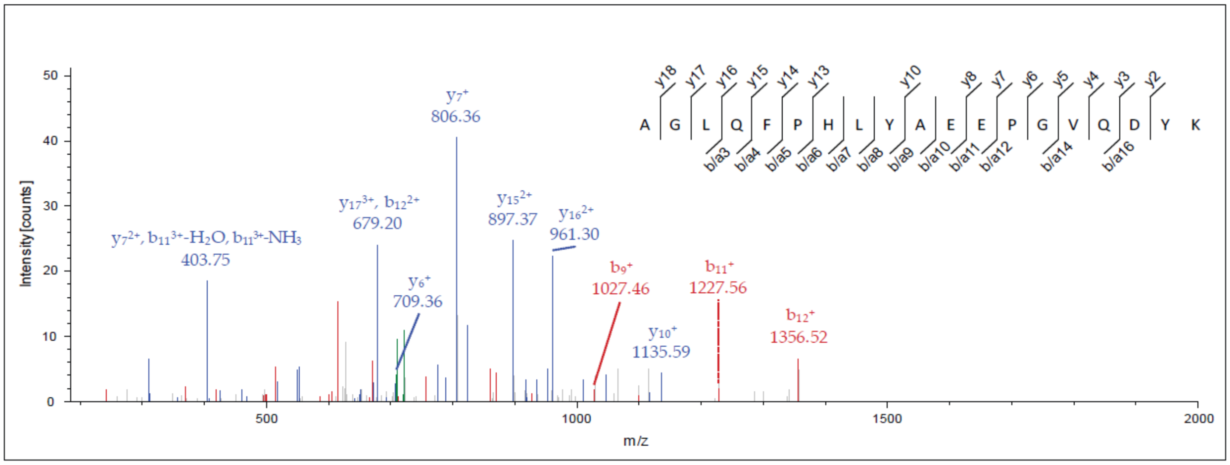

Supplementary Figure 13. The MS/MS spectrum that identifies the peptide AGLQFPHLYAEEPGVQDYK in *BLOC1S3* intronic smORF. The IonScore for this peptide is 48 and the expectation Value is 3.4E-004. The monoisotopic (m/z) is 721.35779 Da (+1.15 mmu/+1.59 ppm).

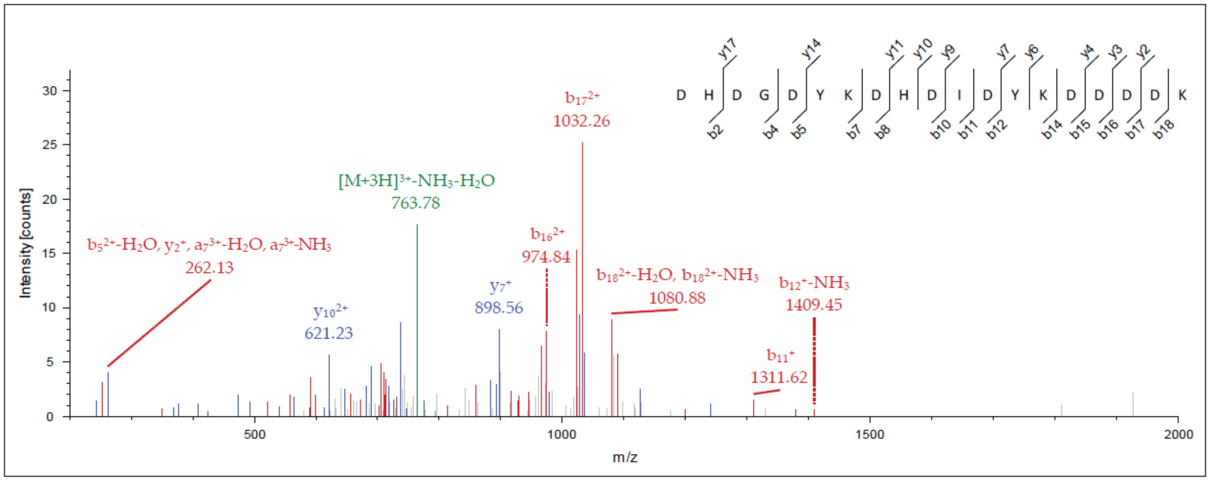

Supplementary Figure 14. The MS/MS spectrum that identifies the peptide DHDGDYKDHDIDYKDDDDK in *BLOC1S3* intronic smORF. The IonScore for this peptide is 49 and the expectation Value is 2.7E-004. The monoisotopic (m/z) is 775.31232 Da (+0.05 mmu/+0.06 ppm).

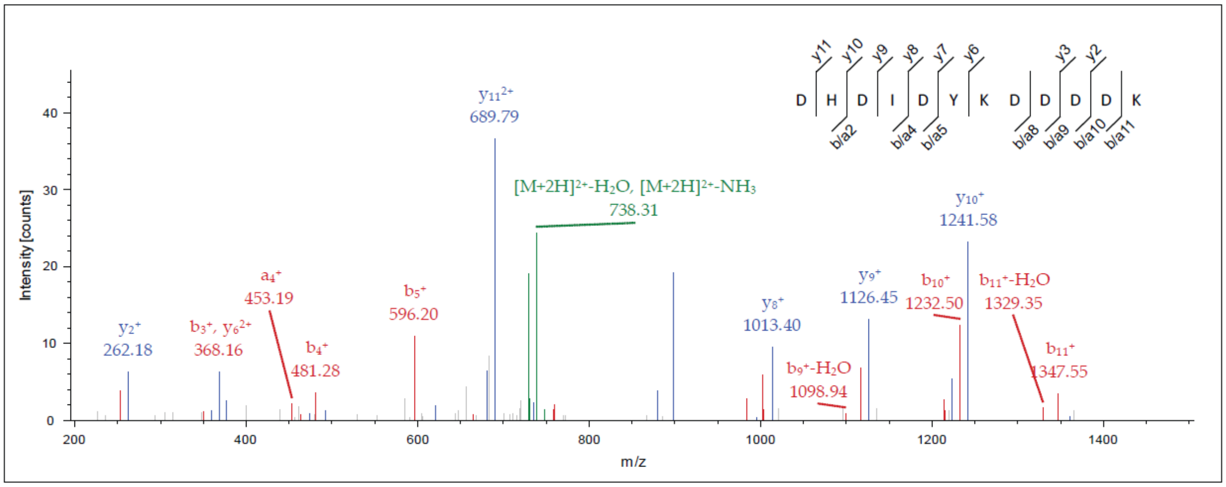

Supplementary Figure 15. The MS/MS spectrum that identifies the peptide DHDIDYKDDDDK in *BLOC1S3* intronic smORF. The IonScore for this peptide is 61 and the expectation Value is 1.8E-005. The monoisotopic (m/z) is 747.30530 Da (+0.29 mmu/+0.39 ppm).

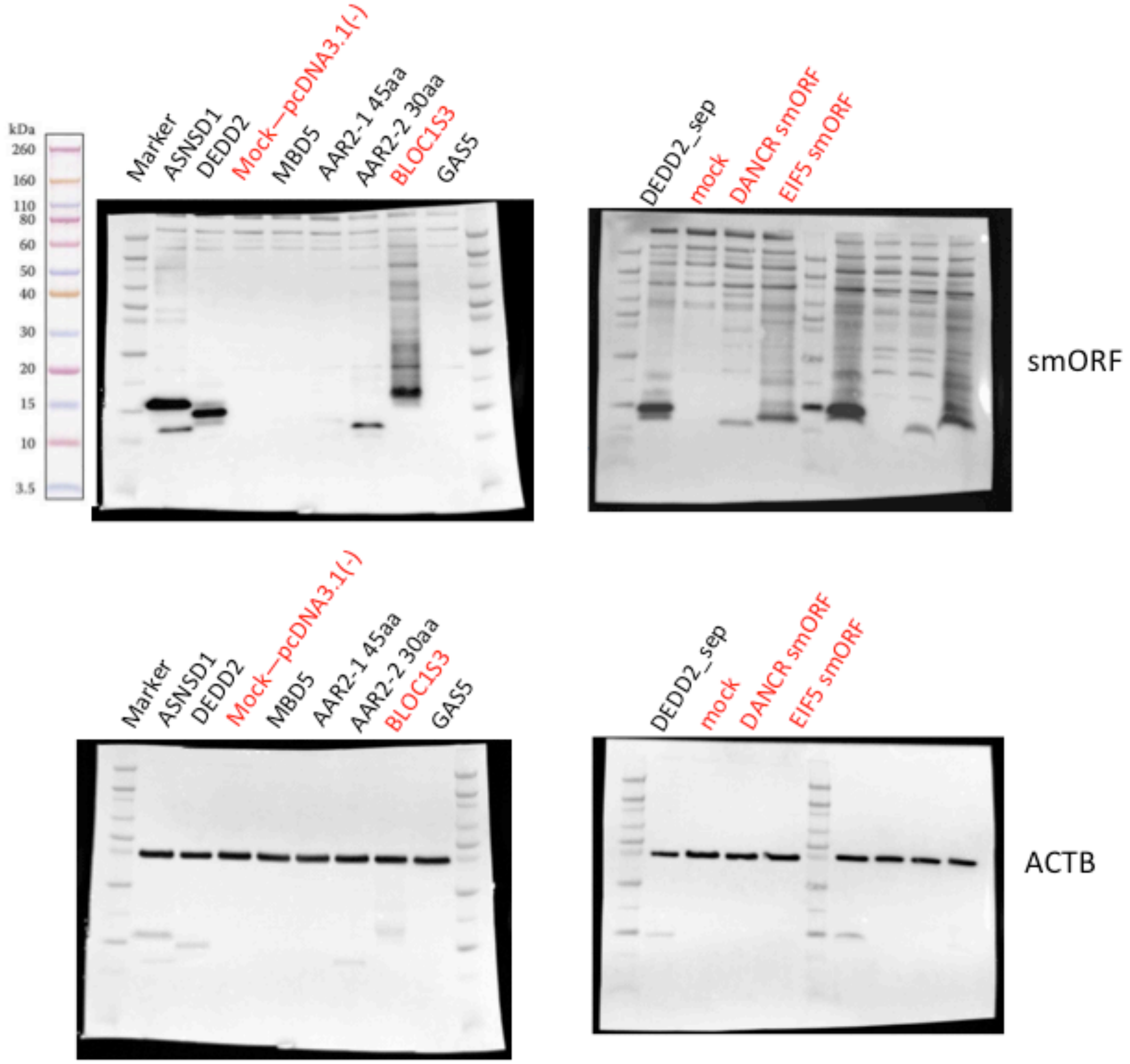

Supplementary Figure 16. The full, uncropped versions of all western blot images.
